# DNA Adenine Methylation Is Required to Replicate Both *Vibrio cholerae* Chromosomes Once per Cell Cycle

**DOI:** 10.1371/journal.pgen.1000939

**Published:** 2010-05-06

**Authors:** Gaëlle Demarre, Dhruba K. Chattoraj

**Affiliations:** Center for Cancer Research, National Cancer Institute, Bethesda, Maryland, United States of America; Stanford University, United States of America

## Abstract

DNA adenine methylation is widely used to control many DNA transactions, including replication. In *Escherichia coli*, methylation serves to silence newly synthesized (hemimethylated) sister origins. SeqA, a protein that binds to hemimethylated DNA, mediates the silencing, and this is necessary to restrict replication to once per cell cycle. The methylation, however, is not essential for replication initiation per se but appeared so when the origins (*oriI* and *oriII*) of the two *Vibrio cholerae* chromosomes were used to drive plasmid replication in *E. coli*. Here we show that, as in the case of *E. coli*, methylation is not essential for *oriI* when it drives chromosomal replication and is needed for once-per-cell-cycle replication in a SeqA-dependent fashion. We found that *oriII* also needs SeqA for once-per-cell-cycle replication and, additionally, full methylation for efficient initiator binding. The requirement for initiator binding might suffice to make methylation an essential function in *V. cholerae*. The structure of *oriII* suggests that it originated from a plasmid, but unlike plasmids, *oriII* makes use of methylation for once-per-cell-cycle replication, the norm for chromosomal but not plasmid replication.

## Author Summary

Bacteria usually have one chromosome but can have extrachromosomal replicons, called plasmids. Although normally dispensable, plasmids can confer adaptive advantage to cells in stressful environments. Bacteria can also have multiple chromosomes, each carrying essential genes, as in eukaryotes. In all organisms, chromosomes duplicate once before the cells divide so that the daughter cells can receive equal genetic dowry, but this is not usually the case with bacterial plasmids. *Vibrio cholerae*, the causative agent for the disease cholera, has a typical bacterial chromosome like the chromosome of the well-studied bacterium *Escherichia coli* and has a second chromosome with many signatures indicating its origin from a plasmid. Here we show that, in spite of the distinct nature of the two chromosomes, they both duplicate once per cell cycle, and they both require DNA adenine methylation for this purpose. Our study suggests that once-per-cell-cycle replication is a necessary feature of a chromosome in multichromosome bacteria, and provides a paradigm of how methylation could endow extrachromosomal replicons with the capacity to duplicate like chromosomes.

## Introduction

The regulatory potential of canonical DNA sequences can be greatly expanded by epigenetic modifications. Methylation is the most common modification of DNA and is widely used to control many cellular processes [1]. In bacteria, DNA methylation is restricted to adenine and cytosine residues [2], and can facilitate or interfere with DNA-protein interactions, thereby modulating various DNA transactions [3]. Such transactions include gene expression, DNA restriction, DNA mismatch repair, and chromosome replication and segregation [4], [5].

Most of our knowledge regarding the role of methylation in chromosome replication comes from studies in *Caulobacter crescentus* and *Escherichia coli*. In *C. crescentus*, initiation of DNA replication requires the adenines of the GANTC sequences in the origin of replication to be methylated on both the top and bottom strands by the methylase CcrM. How the methylation helps the origin function is not known, although methylation lowers DNA stability [6], [7] and thereby could facilitate origin-opening, an essential step in the replication initiation process. It is also possible that the methylation changes DNA structure to facilitate protein-DNA interactions at the origin [8]. Irrespective of the mechanism, methylation not only controls the timing of initiation but also restricts initiation to once per cell cycle [9]. Following initiation, the hemimethylated sister origins cannot be reused in the same cell cycle, as the CcrM methylase is not synthesized until the end of the replication cycle.

In *E. coli*, the methylase is called Dam and acts on the adenines of GATC sequences, which are particularly frequent in the origin of replication, *oriC*. In this bacterium also the methylation most likely helps in origin-opening [8], [10] but plays a more definite role in restricting the initiation to once per cell cycle [11]. In *E. coli*, immediate reinitiation is prevented, not by delaying the synthesis of the methylase, but by preventing its action through sequestration of hemimethylated sister origins by a hemimethylation-specific DNA binding protein, SeqA [12]. Sequestration renders DNA unavailable to the methylase. The sequestration also allows initiation synchrony whereby the multiple origins that *E. coli* maintains during rapid growth fire nearly simultaneously. It is believed that the sequestration process continues at least until all the origins have fired. This happens in a narrow window of time giving rise to the initiation synchrony phenotype [13]. In the absence of Dam, the newly replicated origins, without their hemimethylation marks, remain indistinguishable from the unreplicated ones. The choice of origin for replication being random, once-per-cell-cycle initiation from each origin is no longer guaranteed. As a result, in *dam* mutants, the initiation becomes asynchronous and cells can have origins that do not fire at all or fire more than once in the same cell cycle. The consequences are the same in *seqA* mutants, because without sequestration, replicated origins also remain competent for reinitiation.

The lack of discrimination between replicated and unreplicated origins can lead to origin incompatibility [14]. If extra copies of *oriC* are introduced as plasmids into wild type (WT) *E. coli*, the plasmid copies do not compete with the chromosomal *oriC* because of sequestration of newly replicated origins. Without sequestration, in *dam* or *seqA* mutants, the plasmid copies remain available for reinitiation, and under selection they can block the growth of cells in which the chromosomal origins did not get a chance to fire. Sequestration-deficient strains are therefore not easily transformed with *oriC* plasmids [15]. Thus, although not normally required, Dam or SeqA can be essential in a competitive situation.

*Vibrio cholerae* has two chromosomes (chrI and chrII). The origin of chrI (*oriI*) shares 58% identity with the *E. coli oriC*, and both have similarly high densities of GATC sites. The origin of chrII (*oriII*) also has a high density of GATC sites but has a second feature of a major class of plasmids: repeated initiator-binding sites (iterons) [16]. The *dam* gene is also essential for *V. cholerae*, although the reason has remained unknown [17]. Our interest in the role of methylation in *V. cholerae* chromosomal replication stems from the fact that although the bacterium is a close relative of *E. coli*, plasmids with either *oriI* or *oriII* could transform WT *E. coli*, but not when it lacked Dam [18]. It remained unclear whether the failure to recover transformants in the case of *oriI* is because the origin could not function or because of competition (incompatibility) with the closely related chromosomal *oriC* [14], [18]. Incompatibility is unlikely the case of *oriII*, since it has little similarity to *oriC*. Moreover, while *oriI* and *oriC* are regulated by the DnaA initiator protein, *oriII* is regulated by its own specific initiator, RctB [19]. The reason for the Dam requirement of *oriII* could thus be for the functioning of the origin itself.

Here we show that *oriC* can be replaced by *oriI* in the *E. coli* chromosome, and in this chromosomal context *oriI* functions without requiring Dam or SeqA. Incompatibility with the chromosomal *oriC* thus remains a satisfactory explanation of the earlier finding of a Dam requirement for *oriI* plasmids [18]. For *oriII*, Dam but not SeqA appears to be required as only fully methylated *oriII* DNA, but not hemi- or un-methylated DNA, could bind efficiently to the *oriII*-specific initiator RctB *in vitro*. Since the binding of RctB is a prerequisite for *oriII* function, this provides an explanation for why Dam is essential for *V. cholerae*, chrII being indispensable. Finally, we show that SeqA is necessary to restrict initiation to once per cell cycle for both *oriI* and *oriII*, as is the norm for chromosomal origins. Although chrII is believed to have originated from a plasmid, our findings of the methylation requirement for its initiation and cell-cycle specific regulation are unprecedented in studies of plasmids [20], [21]. It appears that a plasmid origin acquired methylation to function as a chromosomal origin, thus providing a novel example of origin evolution in bacteria.

## Results

### Dam and SeqA are not essential for replication initiation at *oriI* in *E. coli*

**Figure 1:**
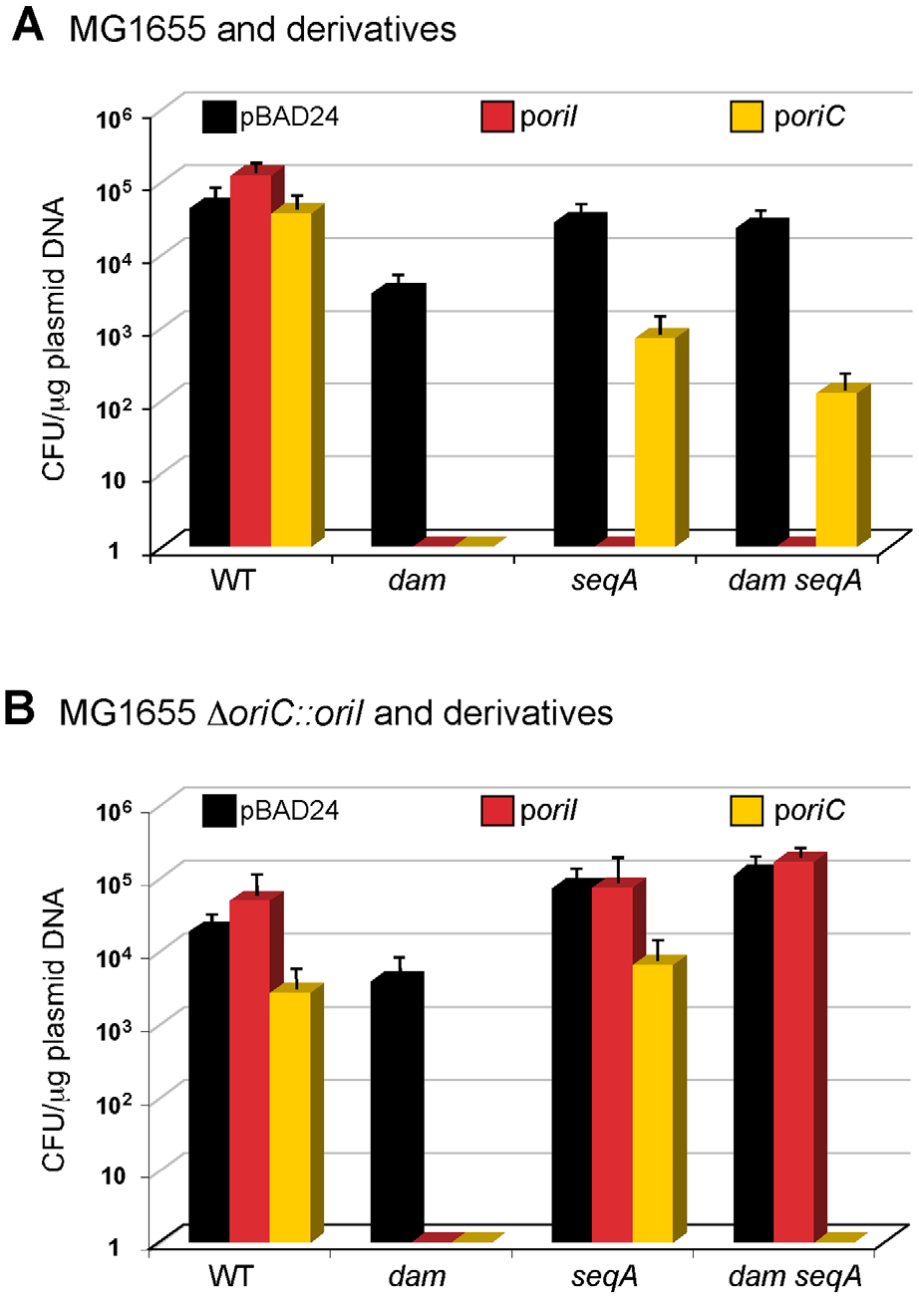
Transformation efficiency of methylated *oriI* and *oriC* plasmids in *E. coli*. (A) The strains used were MG1655 and its *dam16::km*, *ΔseqA10* and *dam16::km ΔseqA10* derivatives, which are abbreviated as *dam*, *seqA* and *dam seqA*, respectively. The plasmids used carried the following origins: pBR322*ori* (in pBAD24; black bars), *oriI* (red bars), and *oriC* (orange bars). The transformation efficiency, expressed as colony forming units (CFU) per µg of DNA, is the average of three independent experiments. The error bars marked here and elsewhere represent one standard deviation of the mean. (B) The starting strain was MG1655*ΔoriC::oriI*, otherwise the details are as in (A).

The *E. coli* origin of replication, *oriC*, does not require *dam* and *seqA* to initiate replication. In contrast, plasmids driven by *oriC* are highly deficient in transformation of *dam* mutants [11]. This is believed to be due to irreversible sequestration of hemimethylated plasmid origins by the SeqA protein after the first round of replication [22]. Indeed, *seqA* and *dam seqA* strains can be transformed by *oriC* plasmids, although the efficiency is lower compared to WT due to incompatibility with the chromosomal copy of the origin [15]. The requirement of *dam* thus is not intrinsic to *oriC* function and appears so only in the plasmid context. The dam requirement of *V. cholerae oriI* has so far been studied only in the plasmid context. However, in contrast to *oriC* plasmids, *oriI* plasmids not only failed to transform an *E. coli dam* mutant but also a *seqA* or a *dam seqA* mutant, raising the possibility that the genes could be essential for *oriI* [11], [18]. We confirmed the plasmid results using *E. coli* MG1655 (BR1703) and its *dam* (CVC1415), *seqA* (BR1704) and *dam seqA* (CVC1424) mutant derivatives. As before, the *dam*, *seqA* and *dam seqA* mutants could not be transformed with an *oriI* plasmid, and only the *dam* mutant could not be transformed with the *oriC* plasmid (Figure 1A). We suggest below that the *oriI* plasmid possibly replicated in the absence of *dam* or *seqA*, which competed out replication from the chromosomal *oriC* and led to inviability of the transformants.

To avoid plasmid-mediated competition (incompatibility), we studied *oriI* by placing it in the *E. coli* chromosome. Using the Red recombineering system, we replaced the minimal *oriC* region with the corresponding *oriI* region (Materials and Methods). The resultant strain, MG1655*ΔoriC::oriI*-*zeo* (CVC1400, Table 1; hereafter called MG1655*ΔoriC::oriI*), could be made *dam* minus by P1 transduction, using *dam-16::aph* (CVC1383) as the source of the mutant *dam* allele [23]. We could also replace the *oriC* region of MG1655*ΔseqA10* with *ΔoriC::oriI* by P1 transduction. The viability of *dam*, *seqA* or *dam seqA* mutant derivatives of MG1655*ΔoriC::oriI* (CVC1401, CVC1416 and CVC1425, respectively) indicates that *oriI* does not require Dam and SeqA for functioning in *E. coli*.

**Table 1:** Bacterial strains.

Strains	Description/relevant characteristics	Reference or source
BR1703 ( = MG1655)	Wild type	[29]
BR1704	BR1703 Δ*seqA10*	[29]
BR2699 ( = DH5α)	*supE44* Δ*lacU169* (φ80*lacZ*'*DM15*) Δ*argF hsdR17 recA1 endA1 gyrA96 thi-1 relA1*	[53]
CVC209	N16961 Str	[46]
CVC769	CVC209 with *parS*-Kn at −90 kb in chrI	R. K. Ghosh
CVC827	CVC209 with *parS*-Kn at 40 kb in chrII	R. K. Ghosh
CVC1060 ( = GM48)	(F^−^) *thr leu thi lacY galK galT ara fhuA tsx dam dcm supE44*	D. Mazel
CVC1061 ( = Π10)	CVC1060 Δ*thyA*::(*erm–pir116*)	[54]
CVC1121	N16961 *hapR^+^ Δdns*	M. Blokesch
CVC1363 ( = Π3813)	B462 Δ*thyA*::(*erm–pir116*)	[54]
CVC1364 ( = β3914)	MG1655 *ΔdapA::(erm-pir) RP4-2-Tc::Mu gyrA462 zei-298*::Tn*10*	[48]
CVC1383 ( = GM3819)	*dam16::aph*	[23]
CVC1394 ( = NM1100)	MG1655 mini-λ Tet	[45]
CVC1400	MG1655 *ΔoriC::oriI-zeo*	This study
CVC1401	CVC1400 *dam16::aph*	This study
CVC1410	CVC209 *ΔseqA*_P_ *-zeo*	This study
CVC1415	MG1655 *dam16::aph*	This study
CVC1416	CVC1400 *ΔseqA10*	This study
CVC1424	MG1655 *dam16::aph ΔseqA10*	This study
CVC1425	CVC1400 *dam16::aph ΔseqA10*	This study
CVC1455	CVC1410 with *parS*-Kn at 40 kb in chrII	This study
CVC1457	CVC1410 with *parS*-Kn at −90 kb in chrI	This study
CVC2003	CVC1121 *ΔseqA*_T_ *-zeo*	This study
CVC2023	CVC209 *Δdam::zeo*/pGD93	This study

To understand why *oriI* and *oriC* behave similarly in the chromosomal context but differently in the plasmid context, we repeated the transformation experiments using MG1655*ΔoriC::oriI* cells as the host. The *oriI* plasmid could now transform the *seqA* and the *dam seqA* derivatives of MG1655*ΔoriC::oriI* efficiently but not the *dam* derivative (Figure 1A and 1B). The failure to transform the *dam* derivative can be attributed to permanent sequestration. In contrast to *oriI*, *oriC* not only failed to transform the *dam* derivative but also the *damseqA* derivative of MG1655*ΔoriC::oriI*. The results can be understood assuming initiation from *oriI* to be more efficient than from *oriC*. Most likely, the weaker *oriC* failed to compete with *oriI* in the chromosome (incompatibility) that led to inviability of the transformants. It is known in *E. coli* that incompatibility problems can be aggravated when the incoming and recipient origins have unequal efficiencies [24].

### Dam and SeqA make initiation synchronous and once-per-cell-cycle for *oriI* in *E. coli*

**Figure 2:**
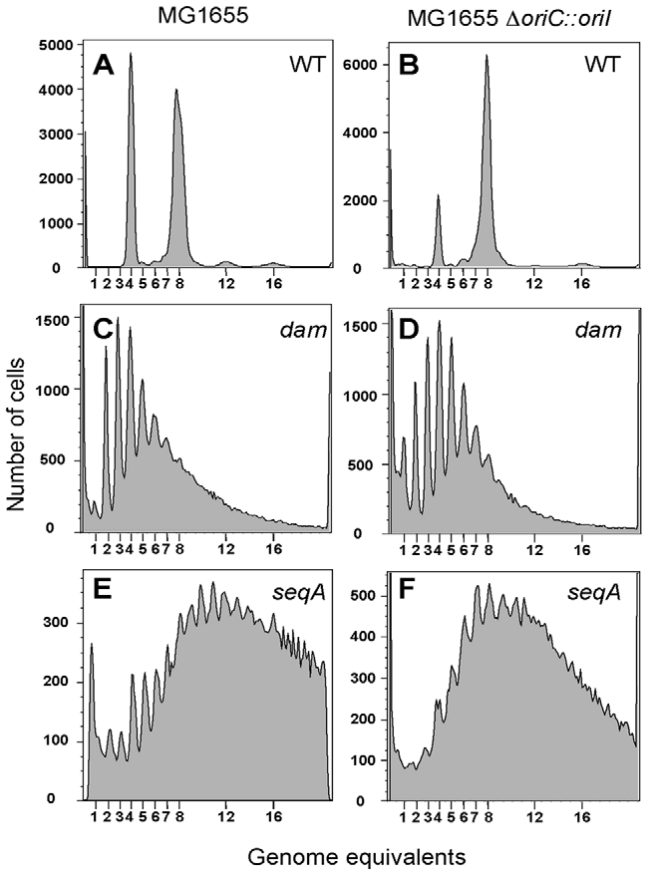
Flow cytometric analysis of DNA content in *E. coli*. The cells used were MG1655 (A) and MG1655*ΔoriC::oriI* (B), and their *dam* and *seqA* mutant derivatives (C, E) and (D, F), respectively. Cells were analyzed after replication-run out in the presence of drugs that inhibit replication initiation and cell division. 100,000 cells were analyzed in each experiment.

*oriI* and *oriC* were further analyzed using flow cytometry [25]. Replication initiation and cell division were blocked by antibiotics rifampicin and cephalexin, respectively, but sufficient time was allowed after drug addition to complete replication elongation (replication run-out). This method provides a measure of the fraction of the population that already initiated replication at the time of drug addition. In LB, after the replication run-out, MG1655 cells were distributed mostly into two populations, one with four and the other with eight full chromosomes (Figure 2A). This indicates that cells were born with four origins and they all fired synchronously once, giving rise to the eight chromosome peak. In the *dam* and *seqA* mutants, cells had a widely varying number of chromosomes indicating asynchronous initiation (Figure 2C and 2E) [22], [26]. There were also cells with more than eight chromosomes indicating that initiation was no longer restricted to once per cell cycle. In the engineered strain, MG1655*ΔoriC::oriI*, replication initiation was synchronous (Figure 2B) but not in its *dam* or *seqA* derivatives (Figure 2D and 2F). The requirements of *dam* and *seqA* for synchronous and once-per-cell-cycle initiation are thus maintained when *oriI* replaces *oriC*.

Compared to the WT, replication initiation was less frequent in *dam* mutants but more frequent in *seqA* mutants in the case of both the origins. As is *oriC*, Dam seems to be playing a positive role and SeqA a negative role in replication initiation from *oriI*.

### Dam is required for initiator binding to *oriII*

It was reported earlier, and we confirmed, that *oriII* plasmids can not transform an *E. coli dam* mutant but can transform a *seqA* mutant [18]. The *oriII* plasmids also failed to transform the *dam seqA* mutant, indicating that irreversible sequestration cannot account for the *dam* requirement. The *oriII* function could not be tested in the chromosomal context, as was done for *oriI*, because attempts to replace *oriC* with *oriII* failed. In any event, incompatibility between *oriC* and *oriII* appears to be an unlikely explanation for the *dam* requirement, as the structure and control elements of the two origins are different [19]. We show below that the reason for the *dam* requirement could be for binding of *oriII* to its specific initiator RctB.

**Figure 3:**
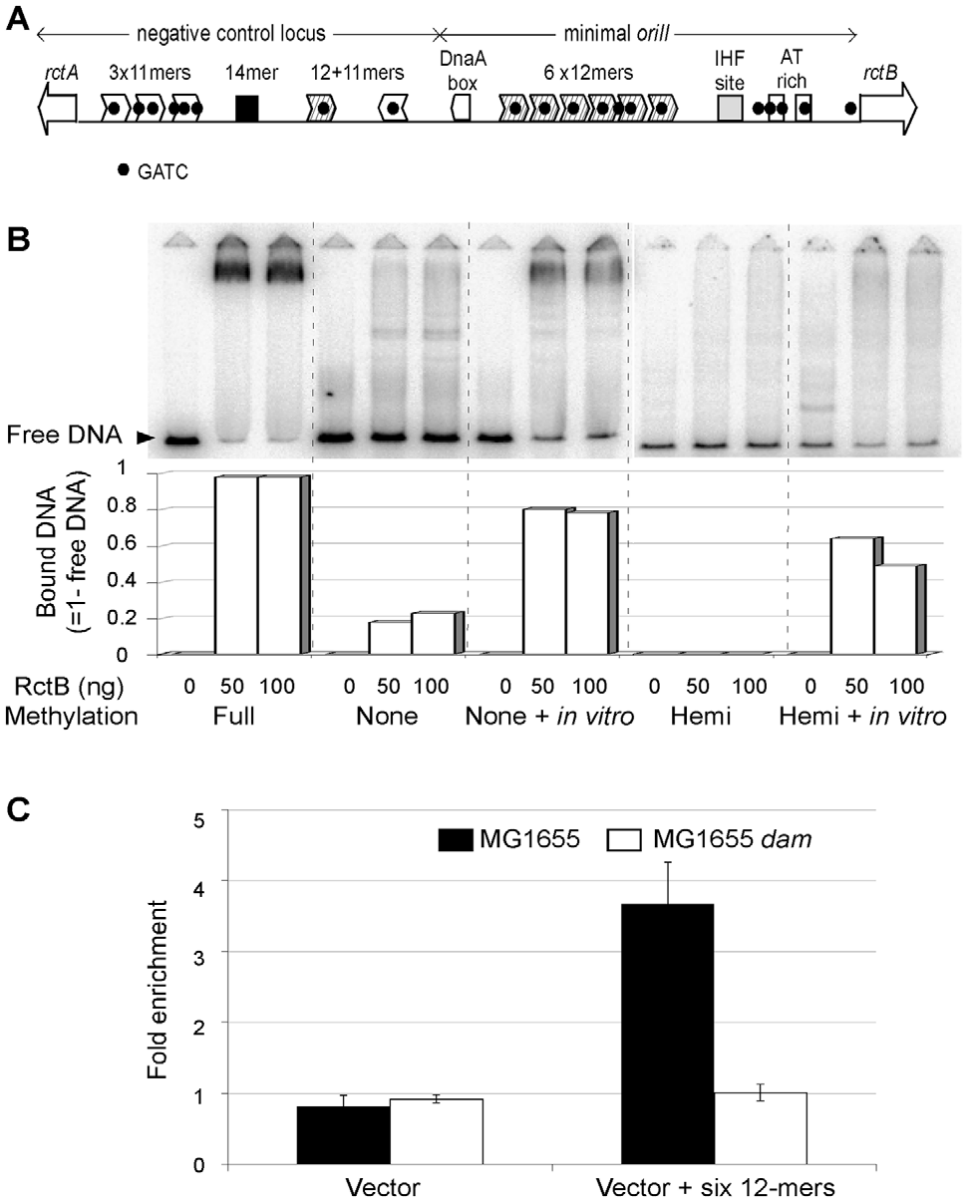
RctB binding to variously methylated *oriII* DNA. (A) A schematic showing the features of the *oriII* region. Two open reading frames, *rctA* and *rctB* (white arrows), border the region. The 11- and 12-mers (white or hatched arrowheads, respectively) are the putative RctB binding sites with GATC sequences (black dots). The origin also has a conserved sequence, 14-mer, and putative binding sites for DnaA (DnaA box) and IHF. (B) Electrophoretic mobility shift assay with fullymethylated DNA (Full), unmethylated DNA (None), the same DNA methylated *in vitro* by Dam (None + *in vitro*), hemimethylated DNA (Hemi) and the same DNA methylated *in vitro* by Dam (Hemi + *in vitro*). The Hemi DNA was methylated on the top strand. RctB amount was 0, 50 or 100 ng per 20 µl binding reaction. The free DNA band (black arrow head) refers to fragments not bound by RctB. The fraction of bound DNA was deduced from the loss of intensity of the free DNA band (Bound DNA = 1-free DNA). (C) Chromatin immunoprecipitation analysis of RctB binding *in vivo*. The precipitation was done with RctB antibody and the cells were either MG1655 (black bars) or its *dam* derivative (CVC1415, white bars), and each carried either the six-12mers (pGD61) or the empty vector (pRLM167). The histogram shows the average of three experiments.

A distinguishing feature of *oriII* is that its putative RctB binding sites, called 11- and 12-mers, all contain a GATC site. This prompted us to test whether methylation of the sites might be important for RctB binding (Figure 3A). We first tested binding to the six tandem 12-mers within the minimal *oriII* by an electrophoretic mobility shift assay. Purified RctB bound efficiently to the 12-mer fragment, when it was fully methylated (Figure 3B). The binding was nearly saturated because most of the DNA molecules were maximally retarded. Binding to hemimethylated DNA, where either the top or the bottom strand carried the methylation marks, and to unmethylated DNA was significantly less. In these cases, most of the bound species appeared as a smear, indicative of weaker binding. The binding improved when the DNA samples were remethylated using Dam *in vitro* (Figure 3B). The binding of RctB to the three 11-mers or to a pair of 12- and 11-mers in the negative-control region of *oriII* was also efficient when the sites were fully methylated (Figure S1A and S1B). Mutating GATC sites to GATG in the 11- or the 12-mer abolished the binding (Figure S1C). These results indicate that full methylation can significantly improve the affinity of RctB to the 11- and 12-mers.

To confirm these results *in vivo*, RctB binding to a plasmid with the six 12-mers was studied in MG1655 or its *dam* derivative by chromatin immunoprecipitation (ChIP), and the immunoprecipitated DNA analyzed by quantitative PCR. Compared to the vector, the plasmid with the 12-mers was preferentially enriched by immunoprecipitation when the DNA samples were from WT cells (Figure 3D). No significant enrichment was obtained when the DNA samples were from the *dam* mutant. These results show the importance of methylation for efficient RctB binding *in vivo*, and therefore, for replication of chrII.

### Dam depletion in *V. cholerae* inhibits *oriII* preferentially

**Figure 4:**
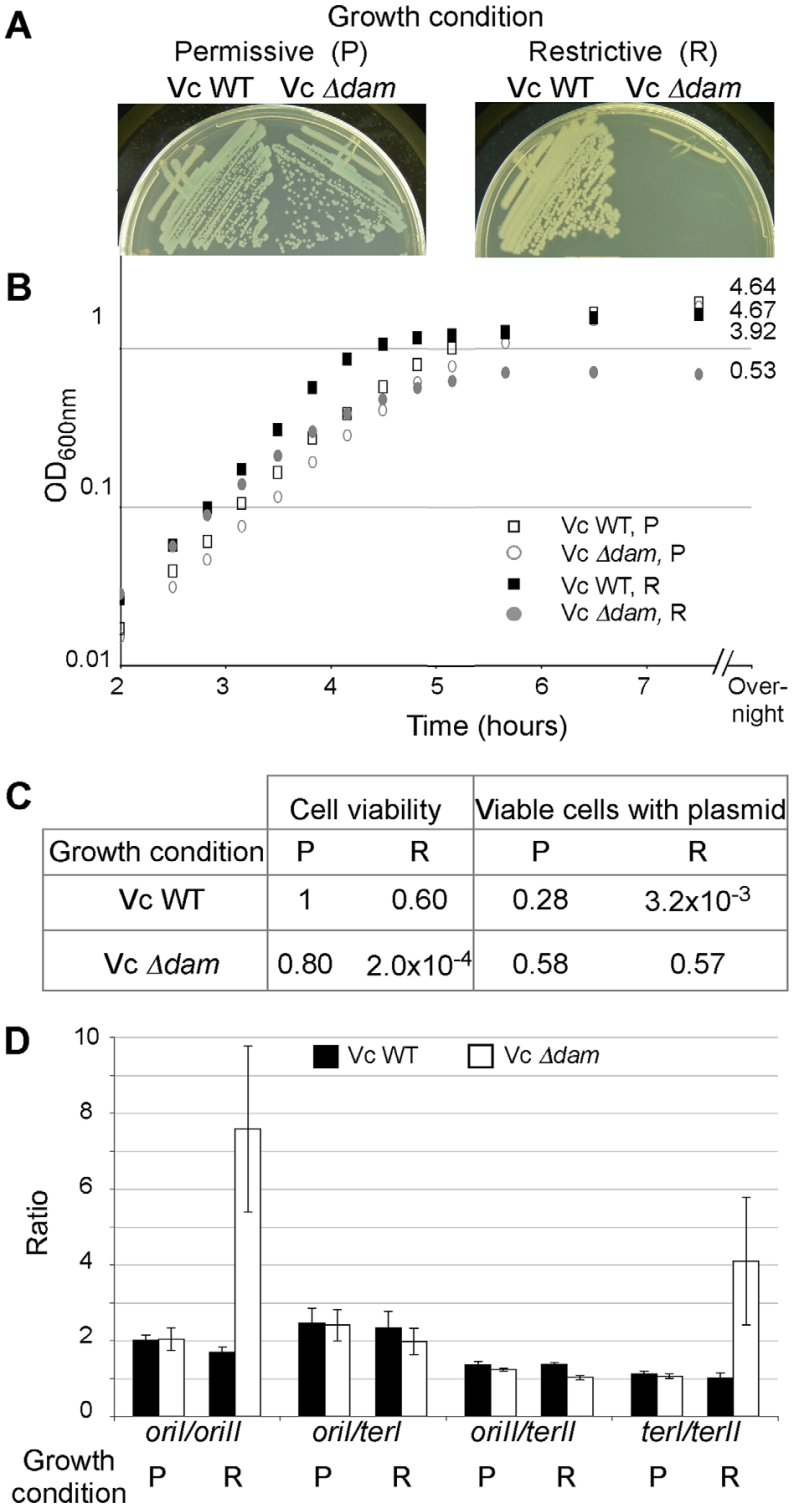
Effect of Dam depletion in *V. cholerae*. Growth of the WT (CVC209/pGD93) and its *Δdam* derivative (CVC2023) on LB plates (A) or in LB broth (B) either under the permissive (P) condition (media with 0.2% arabinose and incubation at 30°C) or under the restrictive (R) condition (media with 0.2% glucose and incubation at 42°C). (C) The viability and the presence of pGD93 after 7.5 hours of growth in LB broth under the permissive and restrictive conditions. To test for the viability and plasmid stability, the cultures were titered on LB plates with or without ampicillin but otherwise under the permissive condition. The values were normalized with respect to those from the WT strain grown under the permissive condition. (D) Analysis of *ori* and *ter* marker frequencies. Cells were from (B) and collected at OD≈0.3. The frequencies of *ori* and *ter* markers in WT (black bars) and Δ*dam* (white bars) cells were compared by qPCR. Crossing point (Cp) values were determined in triplicate and used for calculating the *oriI/oriII*, *oriI/terI* and *oriII/terII* and *terI/terII* ratios.

To test how well the results obtained *in vitro* and in *E. coli* reproduce in the native host, the *dam* gene of *V. cholerae* was deleted in the presence of a complementing plasmid, pTS-P_BAD_ *dam* (pGD93, Table 2). The replication of this plasmid is temperature sensitive and the cloned *V. cholerae dam* gene is under the control of an arabinose-inducible and glucose-repressible promoter, P_BAD_. On LB plates, under the permissive condition (30°C and in the presence of arabinose), the Δ*dam*/pTS-P_BAD_ *dam* strain grew as well as the WT but under the restrictive condition (42°C and in the presence of glucose), single colonies were barely visible (Figure 4A). In LB broth, under the restrictive condition, the mutant grew slower than the WT (with generation times of 27 min and 22 min, respectively), and the growth plateaued to an OD of 0.53 only (Figure 4B). Moreover, the number of viable cells in the mutant culture was only 0.02% of the number of viable WT cells, when initially similar cultures of both were grown for seven and a half hours under restrictive conditions (Figure 4C). The viable cells in the mutant all retained the *dam* complementing plasmid without selection for it. The results thus appear consistent with an earlier report that Dam is essential for *V. cholerae* [17].

**Table 2:** Plasmids.

Plasmids	Description/Relevant characteristics	Reference or source
pBAD24	Cloning vector	[55]
pDS132	Suicide plasmid for allele exchange	[56]
pEM7/Zeo	Cloning vector	Invitrogen
pET22b(+)	Cloning vector	Novagen
pGD55	pBAD24 Flag-*dam*_N16961_ = p*dam*	This study
pGD57	pRLM167::12_mutated_+11 mers	This study
pGD58	pRLM167::12+11_mutated_ mers	This study
pGD59	pRLM167 ::12_mutated_+11_mutated_ mers	This study
pGD61	pRLM167::6×12mers (coordinates 788-934)	This study
pGD63	pBAD24 Flag-*seqA*_N16961_ = p*seqA*	This study
pGD69	p*oriC* (coordinates 4639498 -1497)-*bla*	This study
pGD70	pSW4426T-*ΔseqA*_P_ *::zeo*	This study
pGD79	pSW23-*oriI-zeo*	This study
pGD93	pTS-P_BAD_ *dam* (from pKOBEGA)	This study
pGD114	pEM7-*Δ seqA*_T_::*zeo*	This study
pGD118	pEM7-*Δdam*::*zeo*	This study
pGD121	pDS132-*Δdam::zeo*	This study
pGP704	Cloning vector	[57]
pKOBEGA	Cloning vector; *rep*(ts)	[58]
pRKG256	pGP704::*oriI* (coordinates 2955711-1848) = p*oriI*	R. K. Ghosh
pRLM167	Vector for cloning into a transcription-free zone	R. McMacken
pSW23	Suicide vector	[54]
pSW4426T	pSW23T::*aadA7-araC*-P*_BAD_ccdB*	[48]
pTVC11	pSC101::*rctB*	[59]
pTVC86	pRLM167::3×11 mers (coordinates 291-445)	T. Venkova-Canova
pTVC88	pRLM167::12+11 mers (coordinates 549-718)	T. Venkova-Canova

Under the condition of *dam* depletion, we expected that initiation at *oriII* would decrease more than initiation at *oriI*. This was tested by determining the relative replication efficiencies of the two chromosomes in exponentially growing cells by qPCR. We quantified the amount of DNA at the two origins and the two termini to obtain the ratios *oriI/oriII*, *oriI/terI*, *oriII/terII* and *terI/terII*. Under the restrictive condition, there was a significant increase (4-fold) in the value of *oriI/oriII* and of *terI/terII*, while the values of *oriI/terI* and *oriII/terII* remained unchanged (Figure 4D). These results are consistent with our expectation that compared to chrI, replication of chrII is more dependent on Dam.

### Hemimethylation period is prolonged at *oriI* and *oriII*

**Figure 5:**
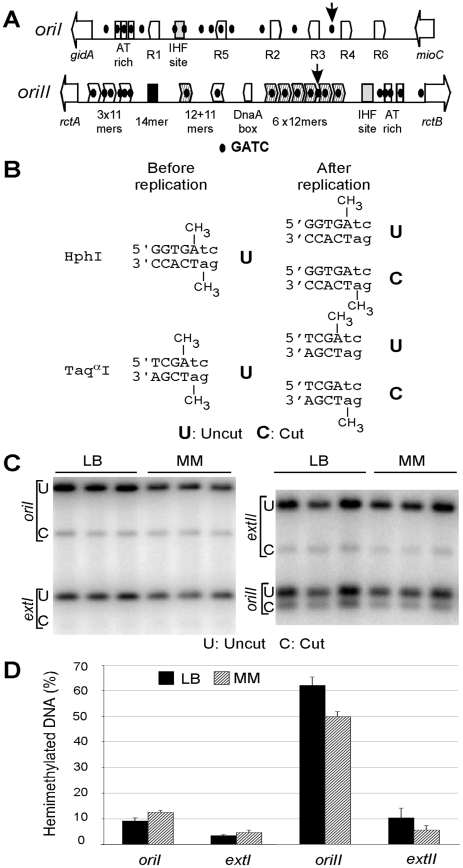
Quantification of hemimethylated DNA in *V. cholerae*. (A) Schematic maps of origin regions of the two *V. cholerae* chromosomes. Both the chromosomal origins (*oriI* and *oriII*) are enriched in GATC sites (black dots). Their relative locations are shown with respect to some other features of the origins. For *oriI*, the features are the flanking genes *gidA* and *mioC*, an AT rich region, DnaA boxes (R1–R6) and an IHF site. The features for *oriII* are described in Figure 3A. Vertical arrows show the two GATC sites studied here for their methylation status. (B) Restriction enzyme names, recognition sequences and their cleavability before and after replication. The recognition sequences are shown in capital letters, and the remainder of the overlapping GATC site is shown in small letters. Prior to replication, these sites are fullymethylated (shown by the attached CH_3_ group on the adenine residues of both the strands) and are uncleavable (indicated by U); passage of the replication fork generates two hemimethylated products, one remains uncleavable but the other becomes cleavable (indicated by U and C, respectively). Thus, the percent of hemimethylated DNA is twice the percentage of cleavable DNA. (C) Probing of the hemimethylation state of GATC sites located either within the origins (*oriI* or *oriII*) or external to the origins (*extI* or *extII*) at about 300 kb away. Autoradiographs of Southern blots show sets of three lanes representing repeat experiments from independent cultures. (D) Quantification of band intensities. The bars represent the mean result of the set of three lanes. The experiments were done in LB (black bars) and in MM (M63 medium with casamino acids; gray bars).

The hemimethylation period, the time to remethylate a GATC site after passage of the replication fork, is particularly prolonged at *oriC* because of the presence of high density of GATC sites within the origin [12]. The prevalence of high density of GATC sites in both *oriI* and *oriII* (Figure 5A) prompted us to examine their hemimethylation period, as was done using asynchronous exponential cultures [27], [28].

We examined the hemimethylation period of a GATC site within the origin and, for comparison, another site external to the origin (about 300 kb away) for each of the chromosomes. In *oriI*, the GATC site chosen is between DnaA boxes R3 and R4, and in *oriII*, it is between the fourth and the fifth 12-mers (arrows, Figure 5A). Total genomic DNA was extracted and digested with restriction enzymes whose recognition sequences overlap a GATC site and whose cleavage is inhibited when the site is fully methylated but not in one of the two hemimethylated sister sites, generated by passage of the replication fork (Figure 5B). The fraction of hemimethylated (cut) DNA at each of the origin sites was significantly higher than at the external sites (Figure 5C). The values were 11±3% and 56±8% for *oriI* and *oriII*, respectively, while at the external markers they were 4±0.8% and 8±3%, respectively (Figure 5D). The results indicate that as in *E. coli*, the hemimethylation period is prolonged at the two *V. cholerae* origins but the duration of the period can be significantly different for the two.

From the *E. coli* paradigm, we expected that SeqA would be required to prolong the hemimethylation periods at both the origins [22]. To test for the requirement, a partial in-frame deletion of *seqA* was made where the deleted region was substituted with a zeocin drug-resistance cassette, maintaining the *seqA* reading frame (Figure S2A). The resulting gene was called Δ*seqA*_P_ and the strain CVC1410. Replication run-out experiments indicated that initiation of one or both the chromosomes has become asynchronous (Figure S2B), and in this respect, *V. cholerae* appears to be similar to *E. coli* (Figure 2A and 2E) [22].

**Figure 6:**
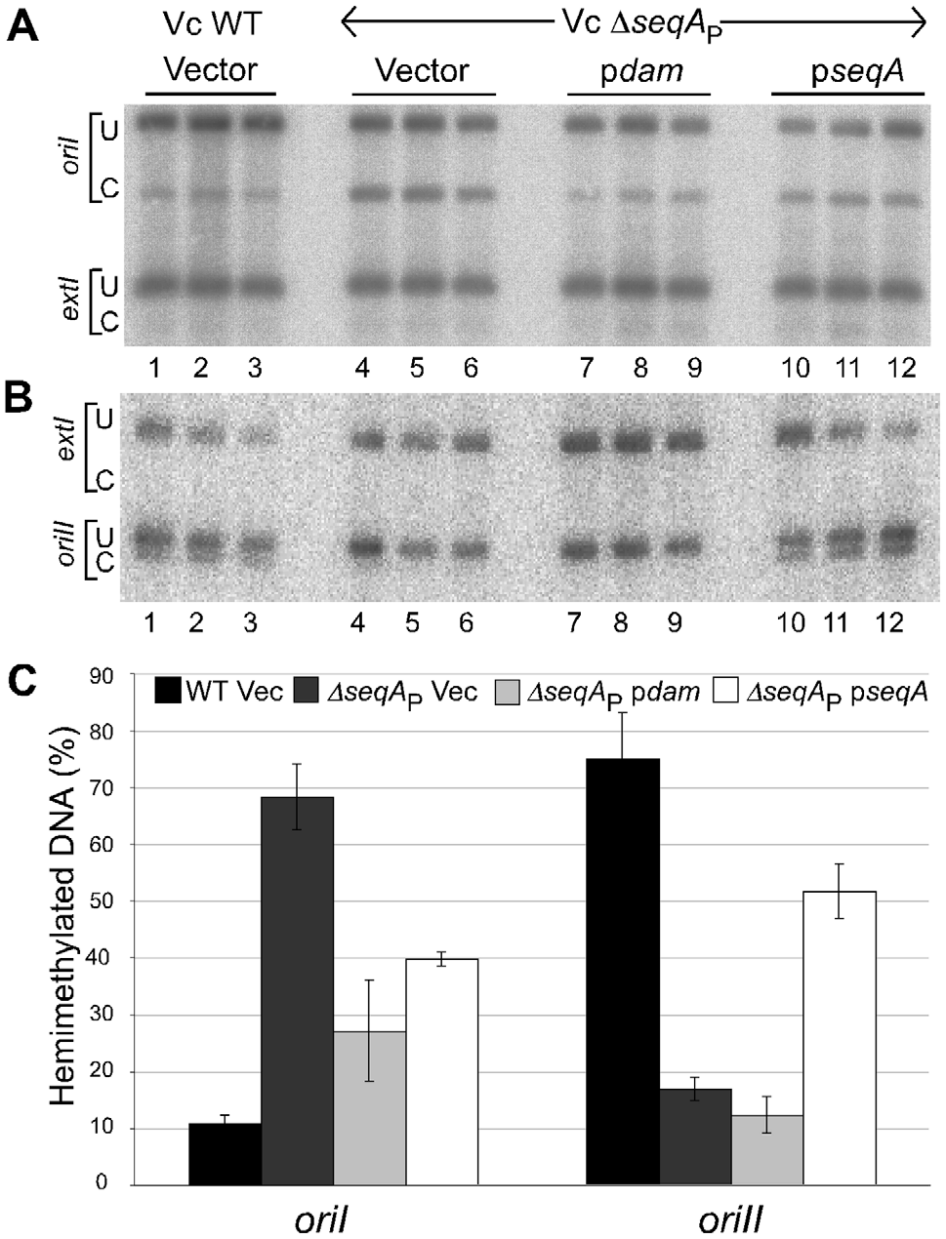
Quantification of hemimethylated GATC sites in WT and Δ*seqA*_P_ strains of *V. cholerae*. Autoradiographs of Southern blots of chromosomes I (A) and II (B). (C) Quantification of band intensities from (A) and (B). The analysis was done in LB in WT (CVC209/pBAD24; black bars), and in a Δ*seqA*_P_ mutant (CVC1410) either with pBAD24 (Vec, dark gray bars) or with a plasmid overexpressing *V. cholerae dam* (p*dam* = pGD65, light gray bars) or *V. cholerae seqA* (p*seqA* = pGD63, white bars). Other details are same as in Figure 5.

For the GATC site tested in *oriI*, the fraction of hemimethylated DNA increased from 11% in WT to 68% in Δ*seqA*_P_ (Figure 6A and 6C). Providing Dam or SeqA from a plasmid in the Δ*seqA*_P_ background decreased the fraction of hemimethylated DNA. The decrease by providing excess of Dam was expected because it converts hemimethylated DNA to fully methylated DNA. The increase in the absence of SeqA and decrease in its presence were unexpected, if SeqA were responsible for prolonging the period. The *seqA* plasmid did not change the period significantly in the WT background (Figure S3). The results indicate that it is the absence of SeqA that causes the increase of hemimethylated *oriI* DNA, a result opposite to that found for *oriC* [29]. The behavior of *oriII* was similar to that of *oriC*: The fraction of hemimethylated DNA decreased from 75% in WT to 17% in *ΔseqA*_P_ (Figure 6B and 6C). Thus *seqA* effects can be opposite in different origins at specific GATC sites. It remains to be seen whether the results are site-specific or true for the entire origins.

**Figure 7:**
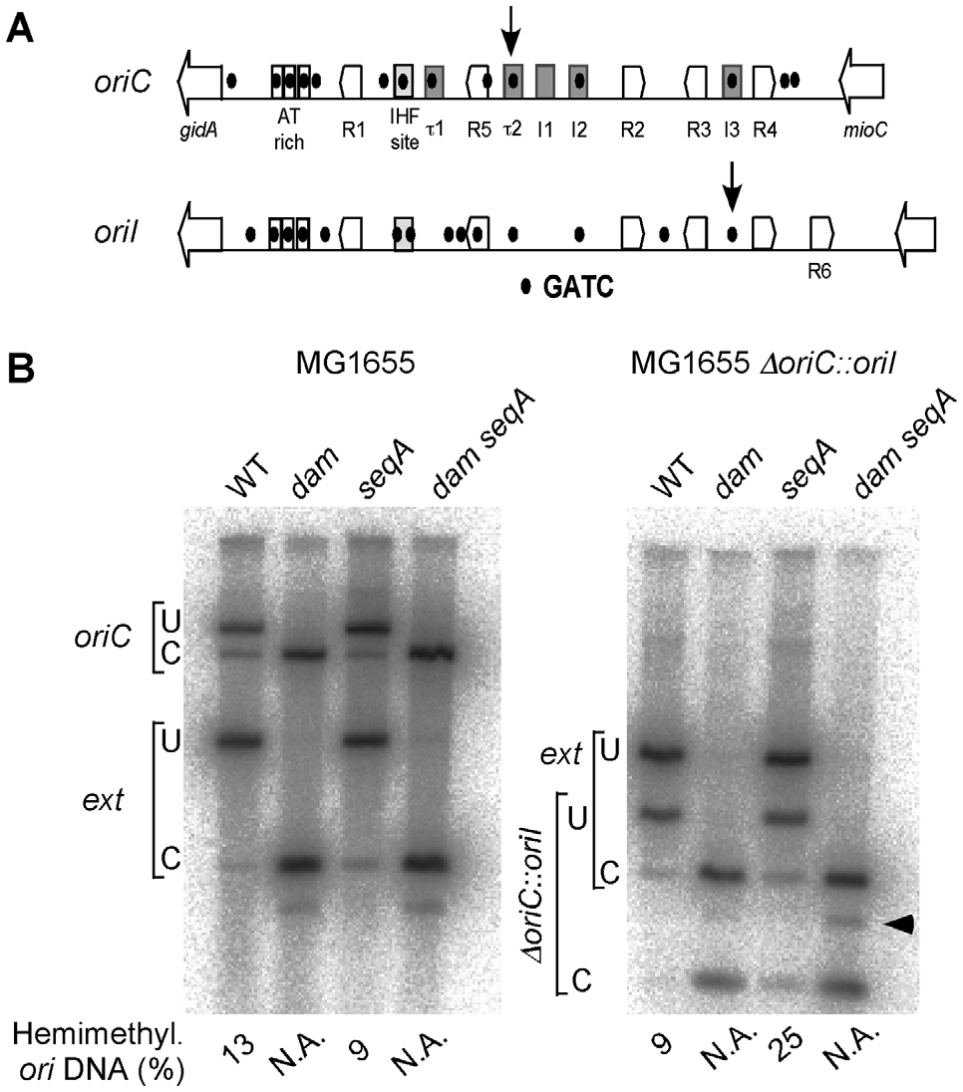
Quantification of hemimethylated GATC sites in *oriC* and *oriI* in *dam* and *seqA* mutants of *E. coli*. (A) Schematic maps of origin regions of *E. coli* chromosome and *V. cholerae* chromosome I. The regions are very similar except that the *V. cholerae* origin (*oriI*) has an extra DnaA box (R6). Other details are described in Figure 5A. The tau and I sites of the *E. coli* origin (*oriC*) that also bind DnaA are yet to be described in *oriI*. The two GATC sites studied here for their methylation states are shown by vertical arrows. (B) The WT either had *oriC* (MG1655) or *oriI* (MG1655*ΔoriC::oriI*). The GATC sites probed were located either within the origins (*oriC* or *oriI*) or external to the origin (*ext*) at about 300 kb away. The same *ext* marker was used for both the strains. The numbers below the figure show the percent of hemimethylated DNA at the origins. N.A. stands for ‘not applicable’; in these lanes the DNA being from a *dam* mutant is unmethylated, and is all cleaved both at the origin and the external markers. An uncharacterized cross-reacting band appears in the *dam* mutants only (arrow head).

The opposite response of the GATC sites tested in *oriI* and *oriII* was also seen in a *V. cholerae* mutant where *seqA* was completely deleted (*ΔseqA*_T_, CVC2003; Figure S4). *oriI* also responded opposite to *oriC* in *E. coli* (Figure 7). While the percent of hemimethylated DNA at *oriC* dropped from 13% in MG1655 to 9% in MG1655*ΔseqA10*, the values at *oriI* increased from 9% in MG1655*ΔoriC::oriI* to 25% in its *ΔseqA10* derivative. These results suggest that the opposite behavior of *oriI* and *oriC* upon *seqA* deletion is intrinsic to the sequence context of the GATC sites tested in the two origins rather than the sequestration machinery of the two bacteria. Thus depending upon the context, SeqA can both shorten and prolong the hemimethylation period of a GATC site.

### SeqA is required for once-per-cell-cycle initiation from both *oriI* and *oriII*

**Figure 8:**
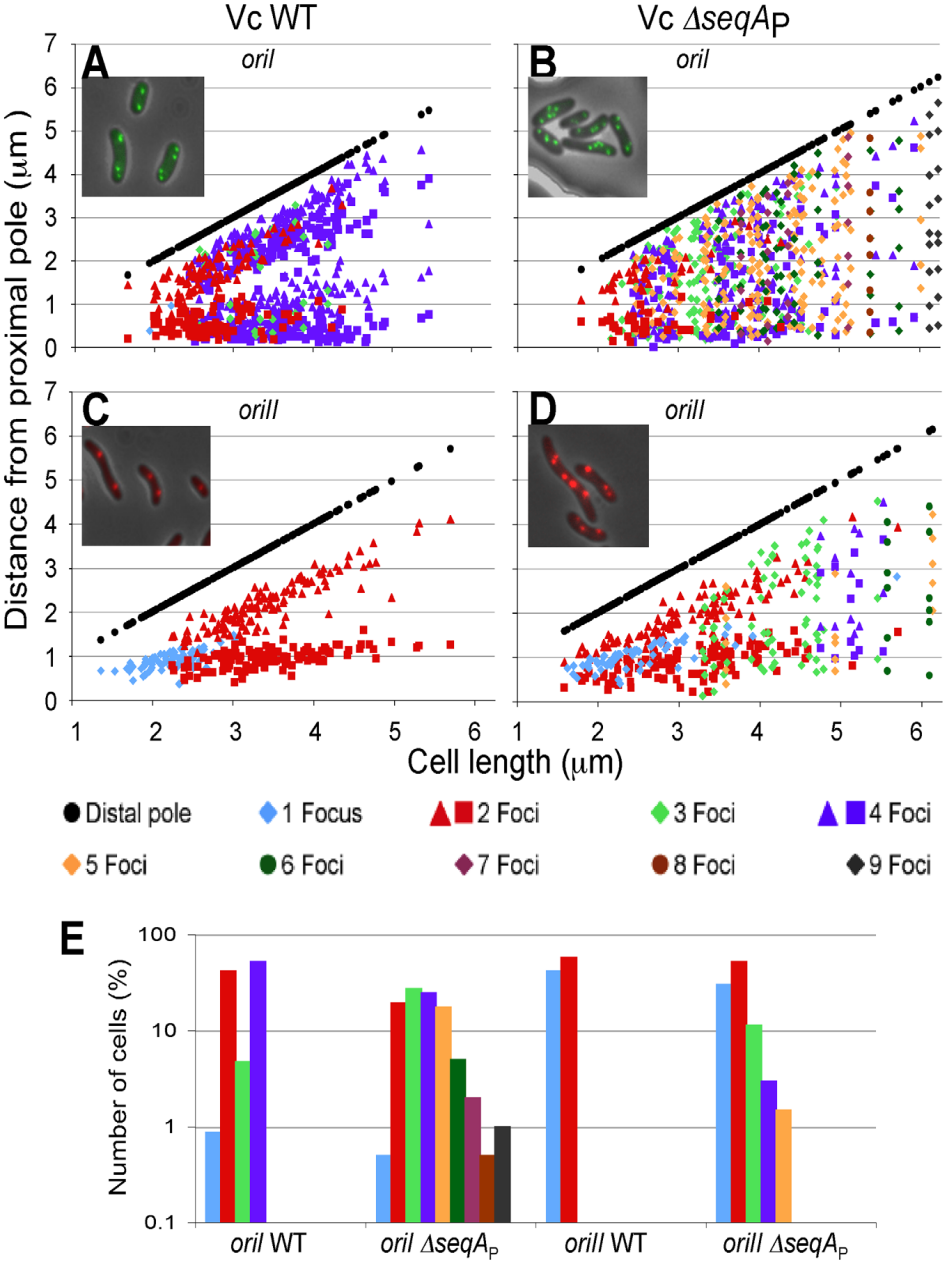
Localization of *oriI* and *oriII* in WT and Δ*seqA*_P_ strains of *V. cholerae*. *oriI* and *oriII* were localized in exponentially growing cells of WT *V. cholerae*: CVC769 for *oriI* (A) and CVC827 for *oriII* (C), and the *seqA*_P_ mutant: CVC1457 for *oriI* (B) and CVC1455 for *oriII* (D). The localization was done using the GFP–P1ParB/*parS* system. *oriI* was marked by inserting P1*parS* at about 90 kb away (counterclockwise to the origin), and *oriII* by inserting P1*parS* at about 40 kb away (clockwise to the origin). Plots show focus positions in cells with one (blue) focus, and two (red), three (light green), four (purple), five (orange), six (dark green), seven (pink), eight (brown) and nine (gray) foci. Focus positions were measured from a pole from which the distance to the nearest focus was smaller, and these (proximal) poles were placed on the abscissa. The other (distal) pole is shown as black circles. 200 cells were analyzed in each experiment. Only cells shorter than 6.25 µm were plotted in all cases. Longer cells accounted for 0 and 0.4% of total cells in WT (A, C), and 4.5 and 6% in the *seqA* mutant (B, D). (E) Distribution of cells vs. the number of foci they contained.

Although a role of SeqA in restraining replication initiation in *V. cholerae* was suggested by the flow cytometry results (Figure S2B), they did not allow us to distinguish whether one or both the chromosomes were affected. We used fluorescence microscopy to follow replication initiation of the two chromosomes individually. The numbers and positions of *oriI* and *oriII* were determined in WT and Δ*seqA*_P_ strains of *V. cholerae* by the GFP-P1ParB/*parS* system [30], [31]. For *oriI* in WT, 94% of the cells had two to four foci and the rest one or three foci, indicating synchronous and once-per-cell-cycle initiation (Figure 8A and 8E). In contrast, only 45% of *ΔseqA*_P_ cells showed this pattern (Figure 8B and 8E). The remaining cells had five to nine foci. The significant increase in the number of cells with odd numbers of foci and more than four foci indicates that initiation is no longer synchronous and no longer limited to once per cell cycle in the absence of SeqA.

The regulation of chrII initiation was also affected. While 100% of the cells in the presence of SeqA showed one to two foci (Figure 8C and 8E), this was true for 83% of the *ΔseqA*_P_ cells (Figure 8D and 8E). The remaining cells showed three to six foci. SeqA thus contributes to synchronous and once-per-cell-cycle initiation of both the chromosomes.

## Discussion

Here we have addressed the role of DNA adenine methylation in replication of the two *V. cholerae* chromosomes. In bacterial replication, adenine methylation can contribute by regulating gene expression, by helping origin opening, and by regulating initiation so that it occurs only once per cell cycle. From the regulatory point of view, the major contribution of methylation is the marking of promoters/origins so that unreplicated DNA can be distinguished from the replicated ones. Newly replicated DNA is uniquely marked with hemimethylated sites that lend themselves to regulation in various ways. In *E. coli*, the newly replicated initiator (*dnaA*) promoter and the origin (*oriC*) are silenced (sequestered) by the SeqA protein, which prevents their reuse for a significant period of the cell cycle. In *C. crecsentus*, the hemimethylated origin and the initiator promoter are also less active but the mechanisms remain unclear. In *V. cholerae*, we show that full methylation of *oriII* promotes initiator binding, providing a new role of the marks in replication initiation, and that SeqA is required for once-per-cell-cycle replication from both the origins (*oriI* and *oriII*), as in the case of *oriC*. By contributing to both initiation and its regulation, methylation thus serves two fundamental requirements for genome maintenance in *V. cholerae*. A comparison of *oriII* to plasmid origins also allowed us to address how a plasmid origin could have evolved to drive a chromosome in a cell-cycle specific fashion. We elaborate on these issues below.

### Methylation and *oriI*

Our work started by questioning the essentiality of Dam and SeqA for functioning of *oriI* since a similar origin, *oriC*, can do without them [18]. We find that the requirements are not real for *oriI* but were imposed due to the use of plasmids to check the origin function. When we replaced *oriC* in the *E. coli* chromosome with *oriI*, making it the only origin in the cell, both the *dam* and *seqA* genes could be deleted (Figure 1). Thus for the functioning of *oriI* and *oriC*, methylation is not essential but it improves chromosomal replication initiation and its control (Figure 2A–2D), including the ability to tolerate extra copies of the origin in trans (Figure 1). In bacteria such as *Bacillus subtilis* that are naturally devoid of the methylation system, *ori* plasmids can exert an inhibitory effect (incompatibility) on chromosomal replication [32]. Methylation thus can help bacterial survival in a competitive situation.

### Methylation and *oriII*

Dam plays a previously unrecognized role for *oriII*. It significantly promotes binding of the chrII-specific initiator, RctB, to the origin, thus possibly serving an essential function (Figure 3). Origin methylation is known to be essential for replication of *C. crescentus* chromosome, and of plasmids P1 and ColV-K30 in *E. coli* [33], [34], [35]. The reason is not clear in these cases, but unlikely to be for initiator binding. The initiator binding sites in these systems lack the sequences required for methylation. In contrast, RctB binding sites have an internal Dam recognition site, and methylation of the sites is required for initiator binding (Figure 3 and Figure S1). Thus, for *oriII*, the mechanism whereby methylation could be essential for its function and, therefore, for the bacterial survival is clear.

### SeqA and once-per-cell-cycle replication of *oriI* and *oriII*

We show that *seqA* is not an essential gene in *V. cholerae* by obtaining viable *seqA* deletion mutants of *V. cholerae*. Although earlier studies suggested the gene to be essential, the finding that both *oriI* and *oriII* could function without SeqA in *E. coli* encouraged us to attempt isolation of the deletion mutants [18], [28]. In a deletion mutant, the number of both *oriI* and *oriII* per cell was found to be greater than in the WT (Figure 8). The overreplication indicates a breakdown of once-per-cell-cycle replication and reveals that SeqA is a negative regulator of replication. The latter was also concluded when the role of SeqA was studied by SeqA overproduction [28]. There was also an increase in the number of cells with odd number of origins for both the chromosomes, indicating loss of initiation synchrony. Thus, SeqA appears to contribute to both once-per-cell-cycle replication and initiation synchrony.

### Hemimethylation periods of *oriC*, *oriI*, and *oriII*

An unexpected finding of this study is that the hemimethylation period of *oriI* and *oriC* changed in opposite ways upon *seqA* deletion: for *oriC* it decreased whereas for *oriI* it increased (Figure 6 and Figure 7). The decrease in the case of *oriC* is expected since SeqA is believed to be the key factor that prolongs the period [22]. A significant increase of the period without requiring SeqA shows that there are other ways to prolong the period, and that SeqA can play an opposite role of shortening the period. The opposite roles of SeqA were seen in isogenic strains of both *V. cholerae* and *E. coli*, suggesting that the reason cannot be due to species-specific factors (Figure 6 and Figure 7). The period also changed in opposite ways for *oriI* and *oriII* in the same *seqA* mutants of *V. cholerae*. SeqA thus has the capacity to both increase and decrease the duration of the period.

SeqA binding to DNA is favored in GATC-dense areas [36], [37]. The density of GATC sites around the diagnostic GATC site happens to be quite different in the three origins. In particular, the diagnostic site in *oriI* is present in a relatively isolated position (Figure 7A). It is possible that the results therein might be site-specific and not representative of the entire origin.

Proteins other than SeqA that interact with origins can also explain the differences in the hemimethylated periods of the origins. DnaA is known to compete with SeqA for binding to some of the sites in *oriC* [38], and can significantly prolong the period even without SeqA [37]. Thus, DnaA is a likely candidate for prolonging the period for *oriI* in the absence of SeqA.

Upon *seqA* deletion, although the hemimethylation period changed oppositely for *oriI* and *oriII*, both the chromosomes over-replicated (Figure 8). The prolongation of the period thus may not always be diagnostic of the role of SeqA in the negative regulation of replication. As stated above, competition with DnaA for *oriI* binding could be another way for SeqA to exert its negative regulatory role [38]. The correlation of the prolongation of the period and the strength of negative regulation was also poor in the case of *oriII*. Although, the period reduced drastically in a *seqA* mutant, the corresponding relaxation of replication was modest (Figure 8). In *oriII*, the negative control is mediated primarily by limiting RctB, which apparently makes the contribution of sequestration to regulation less significant [19].

### Plasmid versus chromosome replication

ChrII has many plasmid-like features including the organization of its origin. Plasmids generally initiate their replication randomly in the cell cycle and control it independently of the chromosome [16], [20], [31], [39]. Plasmid copy number can vary among individual cells due to replication error and unequal segregation. To maintain the mean copy number, plasmids adjust for fluctuations in copy number by replicating more in cells that receive fewer copies than the mean, and replicating less in cells with more copies than the mean. Thus, once-per-cell-cycle replication is not suited for the maintenance of plasmid copy number. We show here that unlike plasmids, chrII replicates once per cell cycle, like other bacterial chromosomes. The high density of GATC sites of *oriII* is not typical for plasmid origins but is a conserved feature of all sequenced strains of the family *Vibrionaceae* [18]. It appears that the involvement of methylation has rendered functioning of a plasmid-like origin similar to that of a chromosomal origin.

Why does initiation need to be cell-cycle specific for the chromosome? Completion of cell division demands that the septum forming area be cleared of DNA [40]. Plasmids are generally small and have correspondingly short replication elongation periods. Incompletely replicated plasmids are unlikely to cause steric hindrance to cell division for a significant period, unlike incompletely replicated chromosomes [41]. If chrII were to initiate replication randomly in the cell cycle like the plasmids, late-initiating chrII would likely delay cell division and create heterogeneity in cell generation times. *V. cholerae ΔseqA* cells did form elongated cells, indicative of a cell division defect (our unpublished results). One reason for this could be steric hindrance to cell division from late-initiating chrII. We suggest that a chromosome replicating from an origin with a plasmid provenance is subject to selection pressure to make the initiation cell-cycle specific, and the acquisition of methylation sites could allow that.

### Methylation and bacteria with multiple chromosomes

Understanding the role of methylation can also be important for another reason. It has been suggested that one of the common conspicuous features of the two origins being the high density of GATC sites, their methylation could be a mechanism to coordinate the replication between the two chromosomes [18]. Methylation is essential for the viability of bacteria with multiple chromosomes such as *Rhizobium meliloti* [42], *Brucella abortus* [43] and *Agrobacterium tumefasciens* [44] in addition to *V. cholerae* [17]. Although there is no evidence yet for direct communication among the chromosomes for replication initiation in any system, it is possible that in these bacteria methylation could be coordinating the replication to the cell cycle, as is does for *V. cholerae* and possibly other members of the family of *Vibrionaceae*.

## Materials and Methods

### Bacterial strains, plasmids, and media

Bacterial strains and plasmids used in this study are listed in Table 1 and Table 2, respectively. Primers are listed in Text S1. *E. coli* and *V. cholerae* were grown in LB (10 g tryptone +5 g yeast extract +5 g NaCl per liter, pH adjusted with NaOH to ∼7) or M63 medium (KH_2_PO_4_ 3 g + K_2_HPO_4_ 7 g + (NH_4_)_2_SO_4_ 2 g + FeSO_4_ 0.5 mg + MgSO_4_.7H_2_O 0.25 g, pH adjusted with KOH to ∼7) supplemented with 2 mM MgSO_4_, 0.1 mM CaCl_2_, 0.01% thiamine and 0.2% glucose, and additionally 0.1% casamino acids when desired. Antibiotics were used at the following concentrations: ampicillin, 100 µg/ml; chloramphenicol, 25 µg/ml for *E. coli*, 5 µg/ml for *V. cholerae*; erythromycin, 20 µg/ml; kanamycin, 25 µg/ml; spectinomycin, 50µg/ml; tetracycline, 15 µg/ml; and zeocin, 25 µg/ml. Diaminopimelic acid (DAP) was used at 0.8 mM, L-arabinose at 2 or 0.2 mg/ml, IPTG at 100 µM and thymidine at 0.3 mM.

### Recombineering in *E. coli*

To replace *oriC* (coordinates 3923756–3924022) with *oriI* (coordinates 2961130–364), the latter was amplified from DNA of CVC209 by PCR using primers GD113 and GD114. The PCR product was digested with EcoRI and BamHI, and ligated to similarly digested pEM7-Zeo. The resulting plasmid, pGD83, was digested with SacI and BamHI, and the fragment containing the *oriI*-*zeo* region was ligated to a similarly digested vector, pSW23, generating pGD79. The *oriI*-*zeo* region of pGD79 was amplified with primers GD124 and GD125, and the product used to replace *oriC* of CVC1394 by the mini-λ Red recombineering method [45]. The mini-λ prophage was eliminated from the strain by a 30°C to 42°C temperature shift. The resultant strain was called MG1655Δ*oriC::oriI-zeo* (CVC1400), and the replacement was confirmed by sequencing of the origin region. The genomic DNA of the *dam* mutant derivative (CVC1401) was confirmed for the absence of adenine methylation by its resistance to DpnI but not to MboI and BfuCI restriction enzymes (data not shown).

### Flow cytometry

Cultures of *E. coli* were grown in LB to OD_600_≈0.2 and processed for flow cytometry after replication run-out in the presence of rifampicin (150 µg/ml) and cephalexin (10 µg/ml) for three hours as described [46]. The peak fluorescence intensity of an overnight grown *E. coli* culture in M63 + 0.2% glucose medium (without casamino acids) was taken to represent one genome equivalent.

### Electrophoretic mobility shift assay

A fragment with six 12-mers was obtained from pGD61 by digestion with XhoI and NotI.

Fragments with three 11-mers and a pair of 12- and 11-mers were obtained from pTVC86 and pTVC88, respectively, by digestion with XhoI and BamHI. For methylated and unmethylated fragments, the plasmids were from a *dam* ^+^ (BR2699) and a *dam* ^−^ (CVC1060) strain, respectively. The fragments were gel-purified, dephosphorylated with Shrimp Alkaline Phosphatase (USB Corporation), and end-labeled with 50 µCi [γ-^32^P]ATP (PerkinElmer) by using 30 units of T4 polynucleotide kinase (New England Biolabs) and purified through ProbeQuant G-50 micro columns (GE Healthcare). To obtain hemimethylated DNA, oligonucleotide primers, TVC64 and TVC138 (Sigma-Genosys), were end-labeled and purified as above. The labeled primers were then used for PCR one at a time with methylated DNA as template for one cycle to obtain two populations of hemimethylated DNA, one with methylation on the top strand and the other on the bottom strand. The binding reactions were essentially as described [47].

### *seqA* deletion

A partial deletion of *seqA* was made by deleting codons 51 to 140 and substituting the deleted region with a zeocin cassette maintaining the *seqA* reading frame as follows. The *seqA* gene was amplified from CVC209 by PCR with primers GD87 and GD88. The product was digested with EcoRI and cloned in similarly digested vector, pSW4426T. The resultant plasmid was used as template for PCR with primers GD91 and GD92 to amplify the 5′ end of *seqA*, the plasmid backbone and the 3′end of *seqA*. After digestion with MfeI, a site of which was present within GD91 and GD92 primers, the PCR product was ligated to the zeocin cassette. The cassette was obtained from pEM7-Zeo by PCR, using primers GD89 and GD90 and digested with EcoRI before ligating to the MfeI fragment. The resulting plasmid, pGD70, containing the Δ*seqA*_P_ *::zeo* allele was used to replace *seqA* of CVC209 by the allele-exchange method [48]. The resulting Δ*seqA*_P_ *::zeo* mutant (CVC1410) grew slower than the WT. In LB at 37°C, the doubling times of the mutant was 32±2 min as opposed to 19±2 min for the WT. The Δ*seqA*_P_ *::zeo* allele is called hereafter Δ*seqA*_P_.

The entire *seqA* ORF was also deleted and substituted with the zeocin cassette as follows. First, a kilobase region located downstream the stop codon of *seqA* was amplified by PCR with primers GD228 and GD229, the product digested with EcoRI and BamHI and cloned in a derivate of pEM7-Zeo (pGD111), previously digested with the same enzymes, generating pGD113. pGD111 is essentially same as pEM7-Zeo except that the multi-cloning site upstream of *zeo* is modified to include KpnI and NdeI restriction sites. Next, a kilobase region located upstream of the start codon of *seqA* was amplified by PCR with primers GD230 and GD231, the product digested with KpnI and NdeI and cloned in pGD113, previously digested with the same enzymes, generating pGD114. The flanking regions of *seqA*, now flanking the zeocin cassette, was amplified by PCR with primers GD257 and GD258 and the linear product was introduced by natural transformation in a *hapR^+^* Δ*dns* derivative of N16961 (CVC1121) essentially as described [49], [50]. The transformants were selected for zeocin resistance and checked for the replacement of the *seqA* gene by the zeocin cassette by PCR and DNA sequencing. The resulting Δ*seqA*_T_ *::zeo* mutant (CVC2003) grew as slow as the Δ*seqA*_P_ *::zeo* mutant with a doubling time of 32±2 min. The Δ*seqA*_T_ *::zeo* allele is called hereafter Δ*seqA*_T_.

### *dam* depletion

A complete deletion of the *dam* ORF and its substitution with a zeocin cassette was obtained by the allele-exchange method in the presence of a complementing plasmid, pGD93. The replication of the plasmid was thermo-sensitive and it carried the *V. cholerae dam* under the P_BAD_ promoter. pGD93 was made as follows: the dam gene was amplified by PCR with primers GD72 and GD73, and the product after digestion with EcoRI and KpnI was cloned in pBAD24, previously digested with the same enzymes, generating pGD55. Next, the NdeI-HindIII fragment from pGD55 containing the *dam* gene was cloned in pKOBEGA, previously digested by NdeI and HindIII, generating the pGD93. For allele-exchange, a kilobase region located downstream of the stop codon of *dam* was amplified by PCR with primers GD261 and GD262, and the product after digestion with EcoRI and BamHI was cloned in a derivative of pEM7-zeo, previously digested with the same enzymes, generating pGD117. A 700 bp region located upstream of the start codon of *dam* was amplified by PCR with primers GD263 and GD264, and the product after digestion with KpnI and NdeI was cloned in pGD117, previously digested with the same enzymes, generating pGD118. The zeocin cassette with the flanking regions of *dam* was amplified by PCR with primers GD268 and GD269, and the product cloned as a blunt end fragment in pSW23, previously digested with SmaI, generating the pGD120. The plasmid was digested with SacI and SalI, and the fragment with the zeocin cassette was cloned into pDS132, previously digested also with the same enzymes. The resulting plasmid, pGD121, was used to replace *dam* of CVC209/pGD93. The resulting strain, CVC2023, was confirmed for the replacement of *dam* by the zeocin cassette by PCR and by DNA sequencing. To deplete Dam, single colonies grown in the presence of ampicillin (to select pGD93) and arabinose (to express *dam*) were used to inoculate LB without any drug but containing glucose (to repress *dam* expression) and the cultures were grown at 42°C (to stop plasmid replication).

### Southern blotting

Genomic DNA was isolated from cells of log phase cultures (OD_600_≈0.3), using the Genelute Bacterial Genomic DNA kit (Sigma). For analyzing chrI and *E. coli* DNA, 1 µg of DNA was digested 2 hours with 7.5 or 15 units of HphI (New England Biolabs) at 37°C, and the products resolved in a 1.5% agarose gel. For chrII, the conditions were similar except that Taq^α^I was used at 65°C. The origin probes were prepared by PCR using primers GD36 and GD37 for *ori*I, GD40 and GD41 for *ori*II, GD67 and GD68 for *oriC*, and GD150 and GD151 for *ΔoriC::oriI*. The primers for external markers on the three chromosomes were GD38 and GD39, GD42 and GD43, and GD128 and GD129, respectively. The probes for *ori* and the external markers were made radioactive using the RediPrimeII random primer labeling kit (GE Healthcare) and [α-^32^P] dCTP (PerkingElmer) and mixed separately for the two chromosomes. The band intensities were recorded and quantified as described earlier [46].

### Marker frequency determination

Marker frequency was determined by qPCR using a PTC-200 Peltier Thermal Cycler (MJ Research) and a LightCycler 480 SYBR Green I Master (Roche). Genomic DNA was prepared from log phase cultures in LB with Genelute Bacterial Genomic DNA kit (Sigma), and 313 pg was used in each reaction as template. The primers were used at 0.3 µM each. They were proximal to either *oriI* (GD136 and GD137) or *oriII* (GD156 and GD157) or *terI* (GD142 and GD143) or *terII* (GD140 and GD141) region of the two chromosomes, and were identical to those described [39]. The primer pairs were such that they produced ∼100 to 130 bp fragments in all cases. Cp (crossing point) values were determined and used for calculating the *oriI/oriII*, *oriI/terI*, *oriII/terII* and *terI/terII* ratios. The ratios were normalized to those of a culture grown to stationary phase in supplemented M63 medium (without casamino acids). Mean ratios were obtained from DNA prepared from three cultures, each grown from independent colonies, and each DNA was analyzed in triplicate.

### Chromatin immunoprecipitation

The method was modified from the one described by Lin and Grossman [51]. Briefly, cultures at OD_600nm_ = 0.3 were treated with 1% formaldehyde at room temperature for 30 min. After cell lysis and sonication, RctB complexes were precipitated with antibody against RctB (IP DNA) and Dynabeads-Protein G magnetic beads (Invitrogen), followed by stringent washings (see Text S1 for the detailed ChIP protocol). After reversal of the cross-links by incubation at 65°C overnight, the samples were treated by protease K (Sigma) and then purified with a PCR purification Kit (Qiagen). To quantify the enrichment of RctB binding sites in the IP DNA, 5 µl of 1∶100 dilution of the IP DNA was used to perform locus-specific real-time qPCR with primers GD218 and GD219, specific to the vector backbone of the plasmid carrying the RctB binding sites, and primers GD191 and GD192, specific to a gene in the *E. coli* genome that served as a reference, as described [52].

## Supporting Information

Figure S1 Requirement of adenine methylation for RctB binding to *oriII*. (A) The negative control locus of *oriII* showing the 11- and 12-mers (hatched or white arrowheads, respectively), which are the putative RctB binding sites with GATC sequences (black dots). (B, C) Autoradiographs of EMSA showing RctB binding to methylated or unmethylated DNA. In (B), the DNA fragments contained either the 12+11-mers or the 3×11-mers. In (C), a 170 bp fragment was used containing the 12+11-mer pair but no GATC sequences outside of these two sites. The fragment was also tested when either one or both of its two GATC sites were mutated to GATG. Note that when both the GATC sites were mutated, no retarded band could be seen whether or not the DNA was extracted from *dam* ^+^ or *dam* ^−^ strain. These results are consistent with methylation being important for efficient DNA binding of RctB *in vitro*.
(0.16 MB DOC)

Figure S2 (A) Western blot analysis of extracts from *E. coli* (Ec) and *V. cholerae* (Vc) cells with either an intact or deleted *seqA* gene. The blots were reacted with anti-SeqA_E.coli_ and anti-RctB antibodies. The latter antibody showed a cross reacting band (∼70 kDa) in all cases that was used as a loading control. The cells used were MG1655 (Ec WT) and its isogenic *ΔseqA10* derivative (BR1704), CVC209 (Vc WT_P_) and its isogenic Δ*seqA*_P_ derivative CVC1410, and CVC1121 (Vc WT_T_) and its isogenic Δ*seqA*_T_ derivative (CVC2003). The molecular weights in kDa of protein markers are shown on the left of the autoradiograph. The proteins interacting with the antibodies are named on the right. Note that in the Δ*seqA*_P_ strain, although the SeqA band is missing, a protein of higher molecular weight interacted with the antibody. This is a SeqA-Zeo fusion protein since we deleted the *seqA* gene partially, and the deleted region was substituted with a Zeocin^R^ cassette in-frame. (B) Flow cytometric analysis of DNA contents in *E. coli* and *V. cholerae*. The cells used were as identified in (A) and analyzed when grown to log phase or after replication run out in the presence of drugs that inhibit replication initiation (rifampicin at 150 µg/ml for *E. coli*) or chloramphenicol at 200 µg/ml for *V. cholerae*) and cell division (cephalexin at 10 µg/ml for both bacteria) (Srivastava et al, 2006. *J Bacteriol* 188: 1060). The fluorescence intensity at the first *E. coli* peak after replication run-out was taken to represent four genome equivalents (Figure 2), and this value was used as a reference to scale the abscissa in all other cases, after accounting for the size difference between the two bacterial genomes. 100,000 cells were analyzed in each experiment.
(1.83 MB DOC)

Figure S3 Effect of Dam and SeqA overproduction on the fraction of hemimethylated DNA in *V. cholerae*. Hemimethylation states of GATC sites were probed both in chromosome I (A) and chromosome II (B), located either within the origin (*oriI* or *oriII*) or external to the origin (*extI* or *extII*) at about 300 kb away. Autoradiographs of Southern blots show sets of three lanes representing repeat experiments from independent cultures. (C) Quantification of band intensities from (A, B). The values represent the mean and standard deviations from the set of three lanes. From the *E. coli* paradigm, overexpression of *dam* was expected to decrease the percent of hemimethylated DNA, and it did for *oriI* (from 18 to 4%). The decrease was less for *oriII* (from 54 and 43%). The results of *seqA* overexpression were expected to be opposite to those of dam, but the increase in hemimethylated DNA was significant only at *oriII* (from 54 to 70%). At the external markers, the hemimethylated DNA remained low upon overexpression. Dam and SeqA thus seem to be involved in prolonging the origin hemimethylation period but they affect the two origins differently. For *oriI*, Dam appears to be limiting, not SeqA, and the results are opposite for *oriII*. The longer hemimethylation period and the relative insensitivity to Dam overproduction suggest that *oriII* is more efficiently sequestered than *oriI*.
(0.19 MB DOC)

Figure S4 Comparison of the effects of a partial and a complete deletion of *seqA* (Δ*seqA*_P_ and Δ*seqA*_T_, respectively) on the hemimethylation periods of specific GATC sites of the two *V. cholerae* chromosomes. The WT and Δ*seqA* strains were identical to those used in Figure S2A. Other details are as in Figure S3. In both the deletion strains, the hemimethylation period increased in the case of *oriI* and decreased in the case of *oriII*.
(0.59 MB DOC)

Text S1 Primers and ChIP protocol.
(0.04 MB DOC)
