# Supplementary material for: DNA Adenine Methylation Is Required to Replicate Both Vibrio cholerae Chromosomes Once per Cell Cycle
Source: PLoS Genet. 2010 May 6;6(5):e1000939. doi: 10.1371/journal.pgen.1000939 (PMC2865523; doi:10.1371/journal.pgen.1000939)
Supplement: Figure S1 — Requirement of adenine methylation for RctB binding to oriII. (A) The negative control locus of oriII showing the 11- and 12-mers (hatched or white arrowheads, respectively), which are the putative RctB binding sites with GATC sequences (black dots). (B, C) Autoradiographs of EMSA showing RctB binding to methylated or unmethylated DNA. In (B), the DNA fragments contained either the 12+11-mers or the 3×11-mers. In (C), a 170 bp fragment was used containing the 12+11-mer pair but no GATC sequences outside of these two sites. The fragment was also tested when either one or both of its two GATC sites were mutated to GATG. Note that when both the GATC sites were mutated, no retarded band could be seen whether or not the DNA was extracted from dam + or dam − strain. These results are consistent with methylation being important for efficient DNA binding of RctB in vitro. (0.16 MB DOC) [file pgen.1000939.s001.doc]

**DNA Adenine Methylation is Required to Replicate Both *Vibrio cholerae* Chromosomes Once per Cell Cycle**

**Gaëlle Demarre, and Dhruba K. Chattoraj**

**Figure S1.** Requirement of DNA adenine methylation for RctB binding to *oriII*. (A) The negative control locus of *oriII* showing the 11- and 12-mers (hatched or white arrowheads, respectively), which are the putative RctB binding sites with GATC sequences (black dots). (B and C) Autoradiographs of EMSA showing RctB binding to methylated or unmethylated DNA. In (B), the DNA fragments contained either the 3 x 11-mers or the 12 + 11-mers. In (C), a 170 bp fragment was used containing the 12 + 11-mer pair but no GATC sequences outside of these two sites. The fragment was also tested when either one or both of its two GATC sites were mutated to GATG. Note that when both the GATC sites were mutated, no retarded band could be seen whether or not the DNA was extracted from *dam^+^* or *dam^-^* strain. These results are consistent with methylation being important for efficient DNA binding of RctB *in vitro.*
