# Supplementary material for: DNA Adenine Methylation Is Required to Replicate Both Vibrio cholerae Chromosomes Once per Cell Cycle
Source: PLoS Genet. 2010 May 6;6(5):e1000939. doi: 10.1371/journal.pgen.1000939 (PMC2865523; doi:10.1371/journal.pgen.1000939)
Supplement: Figure S2 — (A) Western blot analysis of extracts from E. coli (Ec) and V. cholerae (Vc) cells with either an intact or deleted seqA gene. The blots were reacted with anti-SeqAE.coli and anti-RctB antibodies. The latter antibody showed a cross reacting band (∼70 kDa) in all cases that was used as a loading control. The cells used were MG1655 (Ec WT) and its isogenic ΔseqA10 derivative (BR1704), CVC209 (Vc WTP) and its isogenic ΔseqA P derivative CVC1410, and CVC1121 (Vc WTT) and its isogenic ΔseqA T derivative (CVC2003). The molecular weights in kDa of protein markers are shown on the left of the autoradiograph. The proteins interacting with the antibodies are named on the right. Note that in the ΔseqA P strain, although the SeqA band is missing, a protein of higher molecular weight interacted with the antibody. This is a SeqA-Zeo fusion protein since we deleted the seqA gene partially, and the deleted region was substituted with a ZeocinR cassette in-frame. (B) Flow cytometric analysis of DNA contents in E. coli and V. cholerae. The cells used were as identified in (A) and analyzed when grown to log phase or after replication run out in the presence of drugs that inhibit replication initiation (rifampicin at 150 µg/ml for E. coli) or chloramphenicol at 200 µg/ml for V. cholerae) and cell division (cephalexin at 10 µg/ml for both bacteria) (Srivastava et al, 2006. J Bacteriol 188: 1060). The fluorescence intensity at the first E. coli peak after replication run-out was taken to represent four genome equivalents (Figure 2), and this value was used as a reference to scale the abscissa in all other cases, after accounting for the size difference between the two bacterial genomes. 100,000 cells were analyzed in each experiment. (1.83 MB DOC) [file pgen.1000939.s002.doc]

**DNA Adenine Methylation is Required to Replicate Both *Vibrio cholerae* Chromosomes Once per Cell Cycle**

**Gaëlle Demarre, and Dhruba K. Chattoraj**


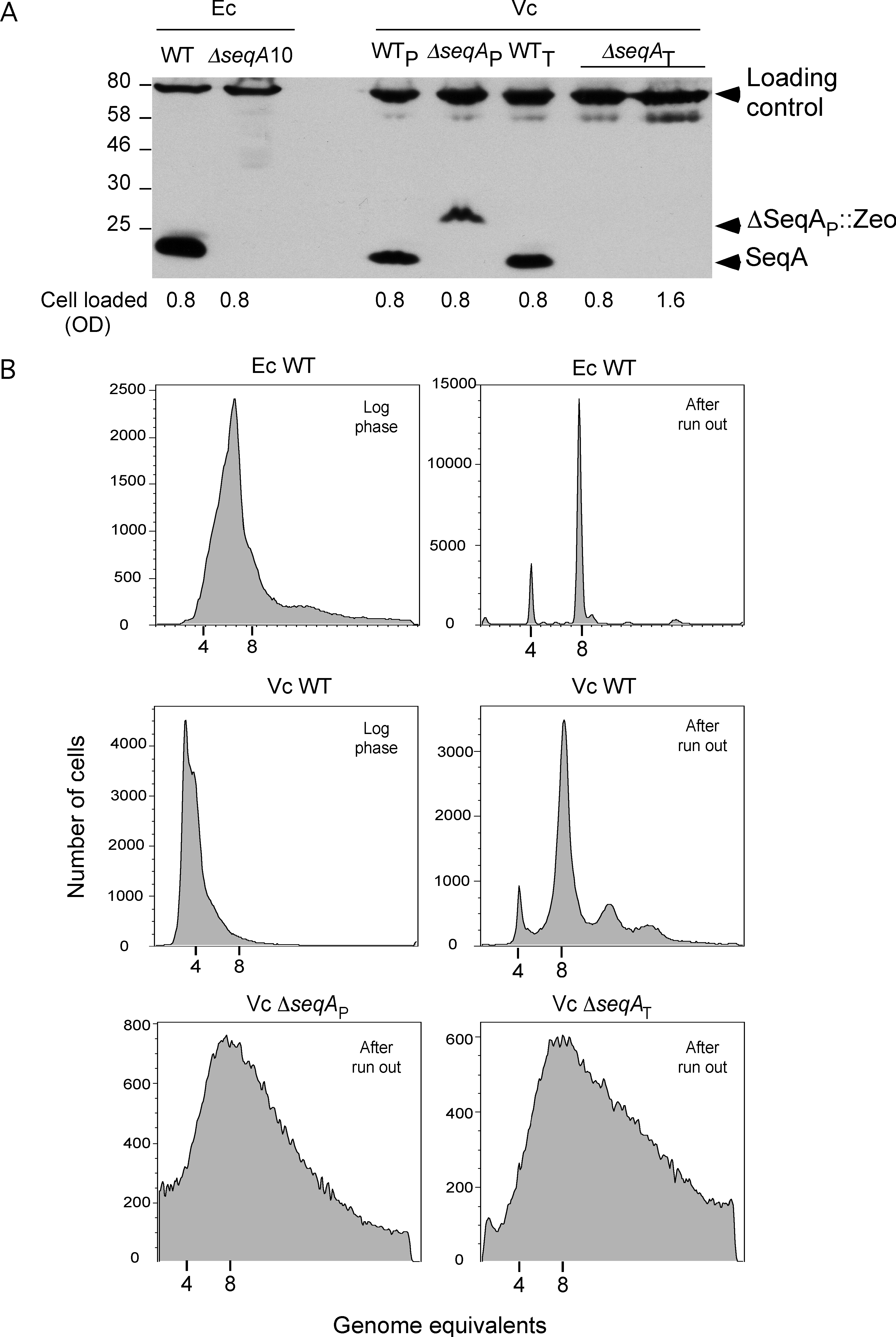


**Figure S2.** (A) Western blot analysis of extracts from *E. coli* (Ec) and *V. cholerae* (Vc) cells with either an intact or deleted *seqA* gene. The blots were reacted with anti-SeqA*_E.coli_* and anti-RctB antibodies. The latter antibody showed a cross reacting band (~70 kDa) in all cases that was used as a loading control. The cells used were MG1655 (Ec WT) and its isogenic Δ*seqA10* derivative (BR1704), CVC209 (Vc WT_P_) and its isogenic Δ*seqA*_P_ derivative CVC1410, and CVC1121 (Vc WT_T_) and its isogenic Δ*seqA*_T_ derivative (CVC2003). The molecular weights in kDa of protein markers are shown on the left of the autoradiograph. The proteins interacting with the antibodies are named on the right. Note that in the Δ*seqA*_P_ strain, although the SeqA band is missing, a protein of higher molecular weight interacted with the antibody. This is a SeqA-Zeo fusion protein since we deleted the *seqA* gene partially, and the deleted region was substituted with a Zeocin^R^ cassette in-frame. (B) Flow cytometric analysis of DNA contents in *E. coli* and *V. cholerae*. The cells used were as identified in (A) and analyzed when grown to log phase or after replication run out in the presence of drugs that inhibit replication initiation (rifampicin at 150 μg/ml for *E. coli* or chloramphenicol at 200 μg/ml for *V. cholerae*) and cell division (cephalexin at 10 μg/ml for both bacteria) (Srivastava et al, 2006*. J Bacteri*o**l 18**8: 1060). The fluorescence intensity at the first *E. coli* peak after replication run-out was taken to represent four genome equivalents (Figure 2), and this value was used as a reference to scale the abscissa in all other cases, after accounting for the size difference between the two bacterial genomes. 100,000 cells were analyzed in each experiment.
