# Supplementary material for: DNA Adenine Methylation Is Required to Replicate Both Vibrio cholerae Chromosomes Once per Cell Cycle
Source: PLoS Genet. 2010 May 6;6(5):e1000939. doi: 10.1371/journal.pgen.1000939 (PMC2865523; doi:10.1371/journal.pgen.1000939)
Supplement: Figure S3 — Effect of Dam and SeqA overproduction on the fraction of hemimethylated DNA in V. cholerae. Hemimethylation states of GATC sites were probed both in chromosome I (A) and chromosome II (B), located either within the origin (oriI or oriII) or external to the origin (extI or extII) at about 300 kb away. Autoradiographs of Southern blots show sets of three lanes representing repeat experiments from independent cultures. (C) Quantification of band intensities from (A, B). The values represent the mean and standard deviations from the set of three lanes. From the E. coli paradigm, overexpression of dam was expected to decrease the percent of hemimethylated DNA, and it did for oriI (from 18 to 4%). The decrease was less for oriII (from 54 and 43%). The results of seqA overexpression were expected to be opposite to those of dam, but the increase in hemimethylated DNA was significant only at oriII (from 54 to 70%). At the external markers, the hemimethylated DNA remained low upon overexpression. Dam and SeqA thus seem to be involved in prolonging the origin hemimethylation period but they affect the two origins differently. For oriI, Dam appears to be limiting, not SeqA, and the results are opposite for oriII. The longer hemimethylation period and the relative insensitivity to Dam overproduction suggest that oriII is more efficiently sequestered than oriI. (0.19 MB DOC) [file pgen.1000939.s003.doc]

**DNA Adenine Methylation is Required to Replicate Both *Vibrio cholerae* Chromosomes Once per Cell Cycle**

**Gaëlle Demarre, and Dhruba K. Chattoraj**

**
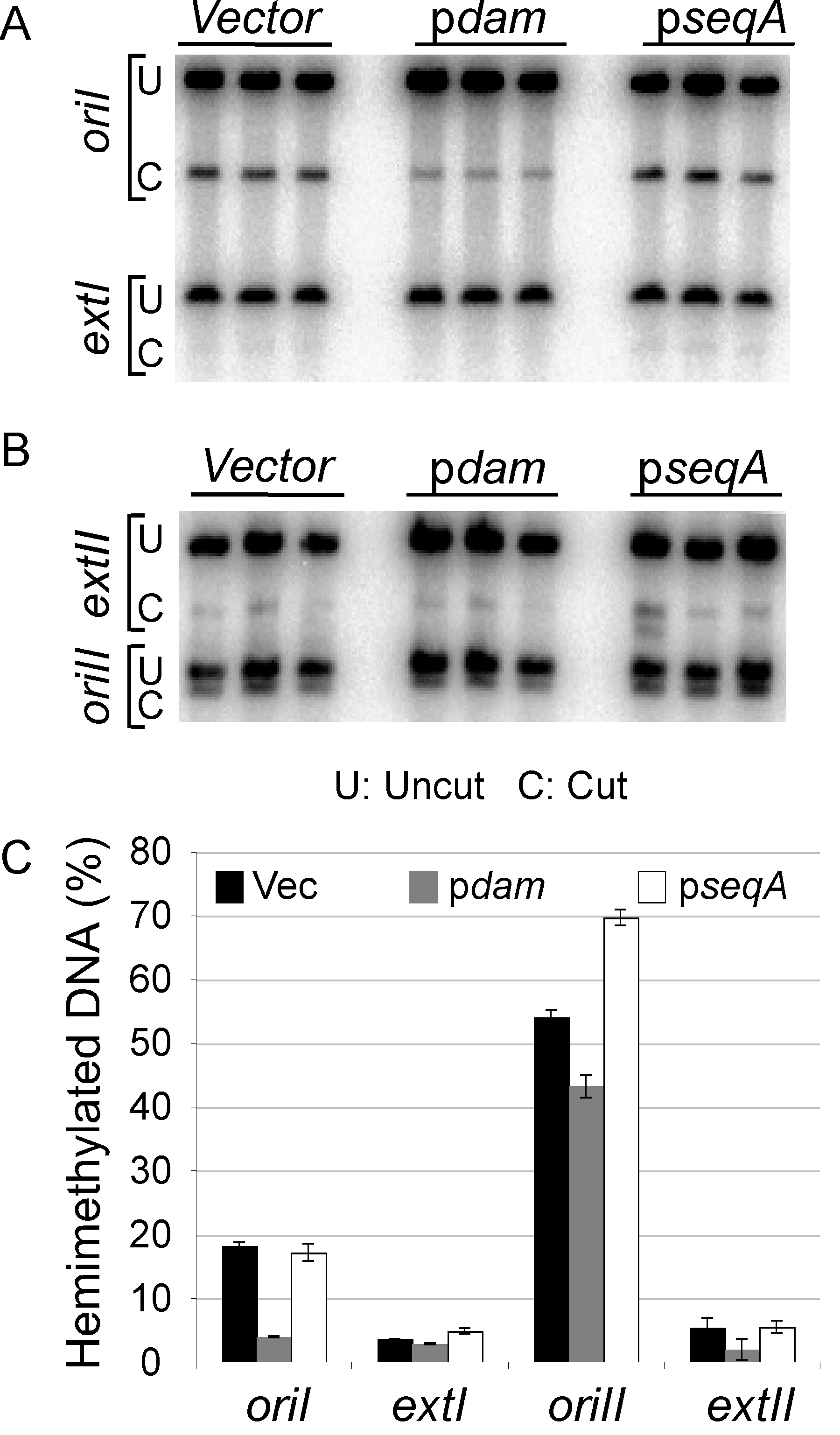
**

**Figure S3.** Effect of Dam and SeqA overproduction on the percent of hemimethylated DNA in *V. cholerae*. Hemimethylation states of GATC sites were probed both in chromosome I (A) and chromosome II (B), located either within the origin (*oriI* or *oriII*) or external to the origin (*extI* or *extII*) at about 300 kb away. Autoradiographs of Southern blots show sets of three lanes representing repeat experiments from independent cultures. (C) Quantification of band intensities from (A) and (B). The values represent the mean and standard deviations from the set of three lanes.

From the *E. coli* paradigm, overexpression of *dam* was expected to decrease the percent of hemimethylated DNA, and it did for *oriI* (from 18 to 4%)*.* The decrease was less for *oriII* (from 54 and 43%). The results of *seqA* overexpression were expected to be opposite to those of *dam*, but the increase in hemimethylated DNA was significant only at *oriII* (from 54 to 70%)*.* At the external markers, the hemimethylated DNA remained low upon overexpression. Dam and SeqA thus seem to be involved in prolonging the origin hemimethylation period but they affect the two origins differently. For *oriI*, Dam appears to be limiting, not SeqA, and the results are opposite for *oriII*. The longer hemimethylation period and the relative insensitivity to Dam overproduction suggest that *oriII* is more efficiently sequestered than *oriI*.
