# Supplementary material for: DNA Adenine Methylation Is Required to Replicate Both Vibrio cholerae Chromosomes Once per Cell Cycle
Source: PLoS Genet. 2010 May 6;6(5):e1000939. doi: 10.1371/journal.pgen.1000939 (PMC2865523; doi:10.1371/journal.pgen.1000939)
Supplement: Figure S4 — Comparison of the effects of a partial and a complete deletion of seqA (ΔseqA P and ΔseqA T, respectively) on the hemimethylation periods of specific GATC sites of the two V. cholerae chromosomes. The WT and ΔseqA strains were identical to those used in Figure S2A. Other details are as in Figure S3. In both the deletion strains, the hemimethylation period increased in the case of oriI and decreased in the case of oriII. (0.59 MB DOC) [file pgen.1000939.s004.doc]

**DNA Adenine Methylation is Required to Replicate Both *Vibrio cholerae* Chromosomes Once per Cell Cycle**

**Gaëlle Demarre, and Dhruba K. Chattoraj**

**
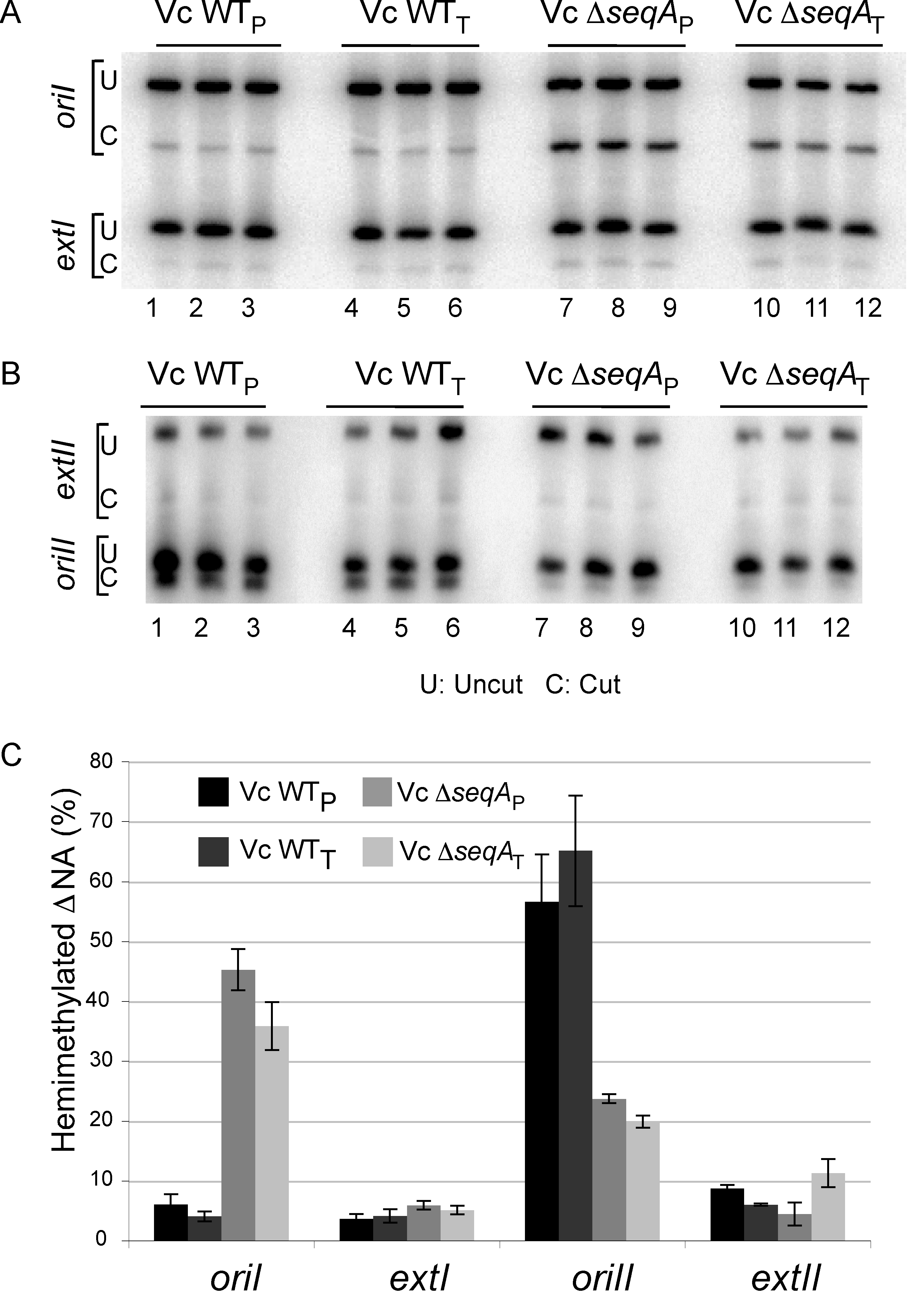
**

**Figure** **S4.** Comparison of the effects of a partial and a complete deletion of *seqA* (Δ*seqA*_P_ and Δ*seqA*_T_, respectively) on the hemimethylation periods of a GATC site on each of the two *V. cholerae* chromosomes. The WT and *ΔseqA* strains were identical to those used in Figure S2A. Other details are as in Figure S3. In both the deletion strains, the hemimethylation period increased in the case of *oriI* and decreased in the case of *oriII.*
