# Supplementary material for: DNA Adenine Methylation Is Required to Replicate Both Vibrio cholerae Chromosomes Once per Cell Cycle
Source: PLoS Genet. 2010 May 6;6(5):e1000939. doi: 10.1371/journal.pgen.1000939 (PMC2865523; doi:10.1371/journal.pgen.1000939)
Supplement: Text S1 — Primers and ChIP protocol. (0.04 MB DOC) [file pgen.1000939.s005.doc]

**DNA Adenine Methylation is Required to Replicate Both *Vibrio cholerae* Chromosomes Once per Cell Cycle**

**Gaëlle Demarre, and Dhruba K. Chattoraj^1^**

**Text S1**

**Primers used in this study**.

The restriction sites used for cloning are underlined.

**Recombineering**

GD113 CCGGAATTCCCTATTCCATGCAGAGCGGC

GD114 CGCGGATCCTGTTTTTTCGATCAAGGAGG

GD124 GGATTCTACTCAACTTTGTCGGCTTGAGAAAGACCTGGGATCCTGTTTTTTCGAT

CAAGGAGG

GD125 TGGGATCGTGGGTTAATTTACTCAAATAAGTATACAGATCGCGATATCGCTAGC

TCGAGCACG

**Hemimethylated DNA**

TVC64 AGATTATCTCGAGCCATTCAGAAGCATGATCAT

TVC138 CTAGGTTGTGCGGCCGCGTGTGAGCACCTTGATCATGCTTAGAA

**Partial *seqA* deletion**

GD87 AAAACTGCAGGAATTCATGAAAACTATTGAGGTTGATGAGG

GD88 AAAACTGCAGGAATTCTTAAATGGAATGGGTAACTTTCTCAATA

GD89 CCGGAATTCGCGATATCGCTAGCTCGAGC

GD90 CGGGAATTCTCAGTCCTGCTC

GD91 CGGCAATTGCGCCGTTTTGGGTGATCACC

GD92 CGGCAATTGCCCTTTCGGCTCAGCAGCAGG

**Total *seqA* deletion**

GD228 CCGGAATTCATTCTGATTCAAGCCTCATAATGC

GD229 CGCGGATCCGTTTAGCCAACGCAATCGGCA

GD230 CCCAATTGCATATGAGATCTTGACTGAGACTCTACG

GD231 CGGGGTACCGGTTGGAATCCGGCATTTGGCG

GD257 GTTTAGCCAACGCAATCGGCA

GD258 GGTTGGAATCCGGCATTTGGCG

***dam* depletion**

GD72 CCGGAATTCGGAGGATTCCGATGGACTACAAGGACGACGATGACAA

AGATATCATGCGATCCACCGGTTTCACC

GD99 CGGGGTACCTTACCTTAGACGATCAAGCTGC

GD261 CCGGAATTCTCTTGTGGGCTGAATGGTGGA

GD262 CGCGGATCCCTGCAGGCTGAGTGCGCGCGCAATC

GD263 CGGGGTACCCTGCAGCGTCATGCGCTATGTTGATCC

GD264 CCCAATTGCATATGCTACTGCTCTAATCGTTGGCC

GD268     AAAACTCGAGAGGCCTGCTGAGTGCGCGCGCAATC
GD269     AAAACTCGAGAGGCCTGGTACCTCAAGCGCAAATGGC

**Southern**

*V. cholerae*

GD36 CCGGAATTCGGAAGCTTGGGTGTTTTCTAACACATTACGC

GD37 CGCGGATCCGCAAGCTTCGGTGATCTTCCTATAGTTATCC

GD38 GCGGGATCCCGTACCGTCTCAGGACGTGG

GD39 CGGGGTACCCAAGCTTCACCAAGCTGTATGGCAGACC

GD40 CCGGAATTCGGAAGCTTCAGGCAGAGTAAGGCTTTGGC

GD41 CGCGGATCCGCAAGCTTCGCATGATCATTTGTTCCTCTAAGC

GD42 GCGGGATCCCGAGCAGCATTCGCTGTTCTGGT

GD43 CGGGGTACCCAAGCTTGGGTTTGGCTGCCCACAGC

*E. coli*

GD67 CCTGGACCGTATAAGCTGGG

GD68 CGTCGCCCATTACGCGTTCCC

GD128 CGCTACCGATAAATCCCTGGG

GD129 GCGAAGTATCGCTCTGCGCC

GD151 GCATAACGCGGTATGAAAATGG

GD150 CCATATAACAAGTTTTAGATCCC

**Marker Frequency Determination**

GD136 CGCCAACCGAGTTTGGATTC

GD137 AAAAAGCGCGTGAGCTTGG

GD140 CACGCAAACAGACCGACACC

GD141 TATCCGCACAGCCTCAGCAA

GD142 GCTTGCGCCGCTTTTAACTG

GD143 CTGAGGCGGATTTGGCACTC

GD156 CTAACTGGGCAGGGATCATTGAT

GD157 ATCACAGCACGAGTACCCGATAT

GD191 CGAGAAACTGGCGATCCTTA

GD192 CTTCATCAAGCGGTTTCACA

GD218 TCAGACTGTGAGCATGAGCCC

GD219 GAAAACCACATCGTCGTTTGATTA

**Chromatin immunoprecipitation (ChIP)**

Cells were grown in 50 ml LB +ampicillin +0.02% arabinose at 37°C. When the cultures reached an absorbance of ≈ 0.3, they were treated with 1% formaldehyde at room temperature for 30 min. The unused formaldehyde was quenched with 125 mM glycine at room temperature for 5 min, and the cells were harvested by centrifugation and washed 3 times with 50 ml of ice-cold PBS. The washed cells were resuspended in 0.5 ml of lysis buffer composed of 10 mM Tris-HCl (pH 8), 20% sucrose, 50 mM NaCl, 10 mM EDTA, 5 mg/ml lysozyme. The cells were incubated at 37°C for 30 min and then treated with 0.5 ml of 2xIP buffer composed of 100 mM Tris-HCl (pH 7.0), 300 mM NaCl, 10 mM EDTA, 2% (vol/vol) Triton X-100, 1 mM PMSF and 50 mg/ml RNase A. The lysate was then sonicated 4 times for 20 sec each in an ice bath to fragment the DNA molecules. Cell debris was removed by centrifugation at 13,000 rpm at 4°C for 10 min using a microcentrifuge, and the resulting supernatant was used as cell extract for immunoprecipitation. The cell extract was first incubated 2 hours at 4°C with 50 μl of Dynabeads Protein G. To immunoprecipitate the protein–DNA complexes, 5 μg of RctB antibody was then mixed with 500 μl of the cell extract. They were then incubated overnight at 4°C, and 50 μl of the Dynabeads Protein G was added to the mixture. After incubation for 5 hours at 4°C, the beads were washed three times with the IP buffer [50 mM Tris-HCl (pH 7.0), 150 mM NaCl, 10 mM EDTA, 1% (vol/vol) Triton X-100], three times with IP-HS buffer [50 mM Tris-HCl (pH 7.0), 500 mM NaCl, 1% (vol/vol) Triton X-100, and 5 mM EDTA], once with LiCl buffer [10 mM Tris-HCl buffer (pH 8.0), 250 mM LiCl, 0.5% (vol/vol) Nonadate P-40, and 1 mM EDTA], and twice with TE buffer [10 mM Tris-HCl (pH 8.0), 1 mM EDTA] in order. After removing the TE buffer, the protein–DNA complexes were eluted first with 100 μl of elution buffer I [10 mM Tris-HCl (pH 8.0), 1 mM EDTA and 1% SDS], then with 150 μl of elution buffer II [10 mM Tris-HCl (pH 8.0), 1 mM EDTA and 0.67% SDS] and incubated overnight at 65°C to reverse the cross-links. After reversal of the cross-links, proteins in the DNA sample were removed by incubation with 40 μg of proteinase K for 2 h at 55°C. The sample was then purified with a PCR purification kit (Qiagen).
